# Antagonistic Roles of Human Platelet Integrin αIIbβ3 and Chemokines in Regulating Neutrophil Activation and Fate on Arterial Thrombi Under Flow

**DOI:** 10.1161/ATVBAHA.122.318767

**Published:** 2023-07-06

**Authors:** Claudia Schönichen, Samantha J. Montague, Sanne L.N. Brouns, James J. Burston, Judith M.E.M. Cosemans, Kerstin Jurk, Beate E. Kehrel, Rory R. Koenen, Fionnuala Ní Áinle, Valerie B. O’Donnell, Oliver Soehnlein, Steve P. Watson, Marijke J.E. Kuijpers, Johan W.M. Heemskerk, Magdolna Nagy

**Affiliations:** Department of Biochemistry, Cardiovascular Research Institute Maastricht (CARIM), Maastricht University, the Netherlands (C.S., S.L.N.B., J.M.E.M.C., R.R.K., S.P.W., M.J.E.K., J.W.M.H., M.N.).; Center for Thrombosis and Hemostasis, University Medical Center of the Johannes Gutenberg-University of Mainz, Germany (C.S., K.J.).; Institute of Cardiovascular Sciences, The Medical School, University of Birmingham, United Kingdom (S.J.M., S.P.W.).; Systems Immunity Research Institute, School of Medicine, Cardiff University, United Kingdom (J.J.B., V.B.O.).; Department of Anaesthesiology and Intensive Care, University Hospital Muenster, Germany (K.J., B.E.K.).; School of Medicine, University College Dublin, Ireland (F.N.Á.).; Department of Haematology, Mater Misericordiae University Hospital and Rotunda Hospital, Dublin, Ireland (F.N.Á.).; Institute for Cardiovascular Prevention, Ludwig-Maximilians-Universität München, Germany (O.S.).; Institute for Experimental Pathology, Center for Molecular Biology of Inflammation, Westfälische Wilhelms Universität, Münster, Germany (O.S.).; Department of Physiology and Pharmacology, Karolinska Institutet, Stockholm, Sweden (O.S.).; Centre of Membrane Proteins and Receptors (COMPARE), Universities of Birmingham and Nottingham, the Midlands, United Kingdom (S.P.W.).; Thrombosis Expertise Centre, Heart and Vascular Centre, Maastricht University Medical Centre^+^, the Netherlands (M.J.E.K.).; Synapse Research Institute, Maastricht, the Netherlands (J.W.M.H.).

**Keywords:** chemokines, integrins, neutrophils, thrombasthenia, thrombosis

## Abstract

**Background:**

Platelets and neutrophils are the first blood cells accumulating at sites of arterial thrombus formation, and both cell types contribute to the pathology of thrombotic events. We aimed to identify key interaction mechanisms between these cells using microfluidic approaches.

**Methods:**

Whole-blood perfusion was performed over a collagen surface at arterial shear rate. Platelet and leukocyte (in majority neutrophil) activation were microscopically visualized using fluorescent markers. The contributions of platelet-adhesive receptors (integrin, P-selectin, CD40L) and chemokines were studied by using inhibitors or antibodies and using blood from patients with GT (Glanzmann thrombasthenia) lacking platelet-expressed αIIbβ3.

**Results:**

We observed (1) an unknown role of activated platelet integrin αIIbß3 preventing leukocyte adhesion, which was overcome by short-term flow disturbance provoking massive adhesion; (2) that platelet-expressed CD40L controls the crawling pattern and thrombus fidelity of the cells on a thrombus; (3) that continued secretion of platelet substances promotes activation of identified neutrophils, as assessed by (fMLP [*N*-formylmethionyl-leucyl-phenylalanine, a potent chemotactic agent and leukocyte activator] induced) [Ca^2+^]_i_ rises and antigen expression; (4) and that platelet-released chemokines activate the adhered cells in the order of CXCL7>CCL5>CXCL4. Furthermore, postsilencing of the platelets in a thrombus suppressed the leukocyte activation. However, the leukocytes on thrombi did no more than limitedly form neutrophil extracellular traps, unless stimulated with phorbol ester or lipopolysaccharide.

**Conclusions:**

Together, these findings reveal a multifaceted regulation of adhesion and activation of neutrophils by platelets in a thrombus, with a balanced role of several platelet-adhesive receptors and a promoting role of platelet-released substances. This multivalent nature of neutrophil-thrombus interactions offers novel prospects for pharmacological intervention.

**Highlights**

- After temporary flow disturbance, leukocytes adhere to and crawl on thrombi with activated platelets.
- The leukocyte (neutrophil) adhesion is enhanced by blocking integrin αIIbβ3 or Glanzmann thrombasthenia platelets.
- Neutrophil activation is mediated by the release of platelet chemokines, causing no more than limited neutrophil extracellular trap formation.

Platelets and neutrophils are the first blood cells accumulating at sites of murine and human arterial thrombus formation and stroke.^1,2^ Both platelets and neutrophils are considered to contribute to the pathology of thrombotic events in arterial and venous thrombosis, myocardial infarction, and stroke.^3,4^ Blood counts of neutrophils were found to correlate with the risk of cardiovascular events.^5,6^ In addition, neutrophil extracellular traps (NETs), large extracellular protrusions of nuclear material, are regularly observed in both venous and arterial thrombi distortion.^7–9^ However, little is still known about the mechanisms of how leukocytes, in particular neutrophils, incorporate into a thrombus and of their early responses.

Previously, the interactions of platelets with neutrophils have been studied in thrombotic, inflammatory, and infection processes.^10–12^ Cell-based studies indicated that these interactions can occur via several adhesive receptors. Described are platelet P-selectin (CD62P) interacting with neutrophil PSGL1 (P-selectin glycoprotein ligand-1);^13^ platelet CD40L recognizing leukocyte CD40^14^; synchronized integrin engagement^15^; and furthermore platelet glycoprotein Ib and JAM-C (junctional adhesion molecule) interacting with integrin αMβ2 on leukocyte populations.^16,17^ This interplay is considered to be important for neutrophils to scan for activated, CD62P-exposing platelets.^18^ Investigators have thus come to the concept of neutrophils screening for platelet-released chemokines, where in particular interleukin 8, CXCL4 (platelet factor 4), CXCL7 (NAP2), and CCL5 (RANTES) are mentioned.^19–21^ Yet, how these manyfold intercellular interactions act in the early setting of thrombus formation is unclear.

Using microfluidics techniques, we have previously assessed the activation processes of platelets and coagulation in shear-dependent thrombus formation.^22,23^ Common platelet activation markers here are rises in cytosolic Ca^2+^, agonist-induced activation of integrin αIIbβ3, CD62P expression, and phosphatidylserine exposure.^24^ Because leukocytes were generally absent during the thrombus buildup in vitro, we hypothesized that platelets may regulate the adhesion of neutrophils and other leukocytes by a tunable set of negative and positive regulatory mechanisms. In the present article, we further developed the microfluidic technique to disclose properties of adhering neutrophils in the setting of arterial thrombosis. We first considered the role of the highest expressed platelet integrin αIIbβ3, using blood from patients with Glanzmann thrombasthenia lacking this integrin.^25^ Subsequently, we examined the effect of flow disturbance and studied the contribution to leukocyte responses of platelet activation markers (CD62P and CD40L) and platelet-released chemokines. Our findings point to a multifaceted regulation of activation by platelets and of mostly neutrophil adhesion on a thrombus.

## MATERIALS AND METHODS

The authors declare that their materials and data are available on reasonable request from the authors.

### Major Resources

This article contains an Extended Materials and Methods section in the Supplemental Material. Please also see the Major Resources Table in the Supplemental Material.

### Blood Collection and Cell Preparation

Blood from healthy volunteers or patients was collected into 10% 129 mmol/L trisodium citrate. All blood donors gave full informed consent according to the Helsinki Declaration. Permission for experiments was obtained from the local Medical Ethical Committees. The approval for in vitro blood cell studies did not allow registration of any medical or sex-based information (except mutations for patients with GT [Glanzmann thrombasthenia] and blood cell counts) of the blood samples. As for common platelet and neutrophil function studies, no sex-related phenotypes are expected, the data from all donors were pooled. Specifically included were 3 patients with Glanzmann thrombasthenia with absent platelet integrin αIIbβ3. Confirmed mutations were for patient 1 (*ITGA2B*, c.2943G>A and 2943G>A, homozygous), patient 2 (*ITGA2B*, c.213C>G and c.2051T>G, compound heterozygous), patient 3 (*ITGA2B*, c.621C>T, homozygous).^26^ Citrated platelet-rich plasma and washed platelets were prepared, as described elsewhere.^27^

### Formation of Heterogenous Platelet Thrombi

Thrombus formation was induced in a parallel-plate flow chamber by perfusion of whole blood over microspots (1.2 mm diameter) of collagen type I at a high wall shear rate of 1000 s^−1^ for 4 minutes.^28,29^ For the formation of type I thrombi without coagulation, citrated blood was recalcified (3.75 mmol/L MgCl_2_ and 7.5 mmol/L CaCl_2_, f.c.) in the presence of D-Phe-Pro-Arg chloromethyl ketone anticoagulant (40 µmol/L). Type II thrombi were formed similarly but using blood that was coinfused with Me-S-ADP (2-methylthioadenosine diphosphate trisodium salt; 1 µmol/L, f.c.) to enhance platelet aggregation. Type III thrombi were produced by coinfusion (9:1, vol/vol) of citrated blood with a coagulation medium (3.75 mmol/L MgCl_2_, 7.5 mmol/L CaCl_2_, 2 pmol/L tissue factor, f.c.).^23^ For thrombi of each type, leukocyte adhesion was introduced by 10 s of flow disturbance, induced by cessation of the perfusion pump, causing a gradual decline of blood flow. This was followed by 2 minutes of flow continuation, after which the blood was removed by rinsing with HEPES buffer pH 7.45, supplemented with 2 mmol/L CaCl_2_. The period of flow disturbance allowed leukocytes present in the 50-mm depth flow chamber to attach to the thrombi that were confined to the collagen microspots.

Platelet activation of thrombi was verified by triple labeling, as appropriate with fluorescein isothiocyanate (FITC)–anti CD62P monoclonal antibody (mAb), PE (phycoerythrin) fibrinogen, and Alexa Fluor 647–annexin A5, as described.^22^ Representative bright-field and requested fluorescence microscopic images were recorded with an inverted Zeiss LSM 510 line-scanning confocal microscope, equipped with 488, 532, and 647 nm laser lines and a ×63 oil objective.^30^ Alternatively, where indicated, an EVOS inverted digital fluorescence microscope was used, provided with bright-field illumination and 470, 531, and 626 nm LEDs (×60 oil objective). Images were analyzed using scripts written in Fiji/ImageJ software.^31^

### Imaging of Neutrophil Activation and NETs

Leukocytes adhered to type I to III thrombi were stained directly after blood perfusion, or after 2 to 24 hours incubation at 37 °C (storage in HEPES buffer containing streptomycin and penicillin, in a humid box). Neutrophil presence and activation markers directly after flow was assessed with Alexa Fluor 647 anti-LFA1 (lymphocyte function-associated antigen 1) or anti-CD15 mAb, FITC anti-CD66b mAb, PE anti-CD162 mAb, and PE anti-CD11b mAb. After staining, the microfluidic chambers were rinsed, and representative microscopic images were acquired. To confirm the activation of platelets and adhered neutrophils, incubated chambers were stained with Alexa Fluor 647 annexin A5 (1 µg/mL), 4′,6-diamidino-2-phenylindole (1 µg/mL), Sytox Green (1 µmol/L), FITC anti-MPO (myeloperoxidase) mAb (2.5 µg/mL), anti-CD66b mAb (5 µg/mL), and anticitrullinated histone-3 antibody (1 µg/mL). The adhered cells were furthermore checked for high responsiveness to fMLP. For confirmation of the ability of NET formation, the neutrophil-containing thrombi were poststimulated with phorbol myristate acetate (PMA) or lipopolysaccharide (50 nmol/L, 1 µg/mL, respectively) and imaged after 4 hours using staining with PE anti-MPO mAb (2.5 µg/mL) and anticitrullinated histone-3 antibody (1 µg/mL), followed by secondary FITC-labeled mAb and 4′,6-diamidino-2-phenylindole (1 µg/mL).

### Cytosolic Ca^2+^ Rises in Single Leukocytes and Platelets

For measurement of single-cell [Ca^2+^]_i_ transients, buffy coats from control blood samples were incubated with 8 µmol/L Fluo-4 acetoxymethyl ester in the presence of pluronic (0.4 µg/mL) for 40 minutes. Leukocyte count and differentiation was determined with a Sysmex-XP300 hematology analyzer. After a centrifugation step, all Fluo-4–loaded leukocytes were resuspended in the double volume of Hepes buffer pH 7.45.

For fluorescence recordings, the Fluo-4–loaded leukocytes (1×10^6^/mL) were perfused over autologous type III thrombi at a shear rate of 200 s^−1^ for 5 minutes. This resulted in adhesion of >98% fluorescent leukocytes (in majority fMLP-responsive, indicative of neutrophils) compared with the nonlabeled leukocytes. Fluorescence changes in microscopic fields were then recorded for 10 minutes at 0.2 Hz, using a Zeiss LSM 510 confocal microscope (488 nm excitation, ×63 oil objective).^30^ The recorded stacks of time series contained both fluorescence and overlay bright-field images. In addition, isolated suspensions of Fluo-4–loaded leukocytes were imaged on coverslip-containing incubation chambers. Where indicated, the coverslips were precoated with immobilized recombinant receptor ligands, perfused with Fluo-4–loaded cells, and, after adhesion, poststimulated with fMLP, CXCL7, CCL5, or platelet releasate (see below).

To measure rises in [Ca^2+^]_i_ in platelets, 20% of washed Fluo-4–loaded platelets were added to autologous blood samples^32^ and flowed over collagen under coagulant conditions in the presence of tissue factor.^23^ Fluorescence changes in the presence or absence of iloprost were video-imaged over time for 10 minutes and pseudoratioed.^32^

To measure rises in [Ca^2+^]_i_ in response to chemokine stimulation, isolated and Fluo-4–labeled leukocytes were incubated with HEPES buffer (control), CXCL7 (100 ng/mL), or CCL5 (100 ng/mL). Alternatively added was the centrifuged releasate from washed platelets (1.5×10^9^/mL) stimulated with thrombin (4 nmol/L) and CRP-XL (collagen-related peptide cross-linked; 3 µg/mL). Images were recorded for 5 to 10 minutes at 0.2 Hz with a Zeiss LSM 510 confocal microscope (488 nm excitation). For a global overview, confocal time stacks of Fluo-4 fluorescence images were analyzed for [Ca^2+^]_i_ transients per region of interest, representing single cells or a thrombus. To assess the activation stage of the adhered leukocytes, videos were observed for the type of oscillatory pattern of fluorescence intensity above resting level (set at *F/F*_*o*_*>*1.15), using rainbow color thresholding. Cells with a Ca^2+^ score of 4 showed multiple, high amplitude rises in [Ca^2+^]_i_; a score of 3 indicated a single, high amplitude rise in [Ca^2+^]_i_; a score of 2 represented low-amplitude rises in [Ca^2+^]_i_; a score 1 was for no fluorescence change above *F/F*_*o*_ 1.15. Fractions of cells per score were integrated to obtain an overall weighted score. For further detailed analysis of single-cell rises in [Ca^2+^]_i_ and movement patterns, see Supplemental Material.

### Statistics

Significance of difference between groups was determined with a nonparametric Mann-Whitney *U* test (2 groups) or a Kruskal-Wallis test (>2 groups), using GraphPad Prism 7 or higher. Scored fractions and classified Ca^2+^ traces were compared by a χ^2^ test. Statistical significance was set at *P*<0.05 and shown as applicable in the graphs. NS *P* values are not shown unless indicated.

## RESULTS

### Suppressed Leukocyte Adhesion to Thrombi by Platelet Integrin αIIbß3 Activation

**Figure 1.**
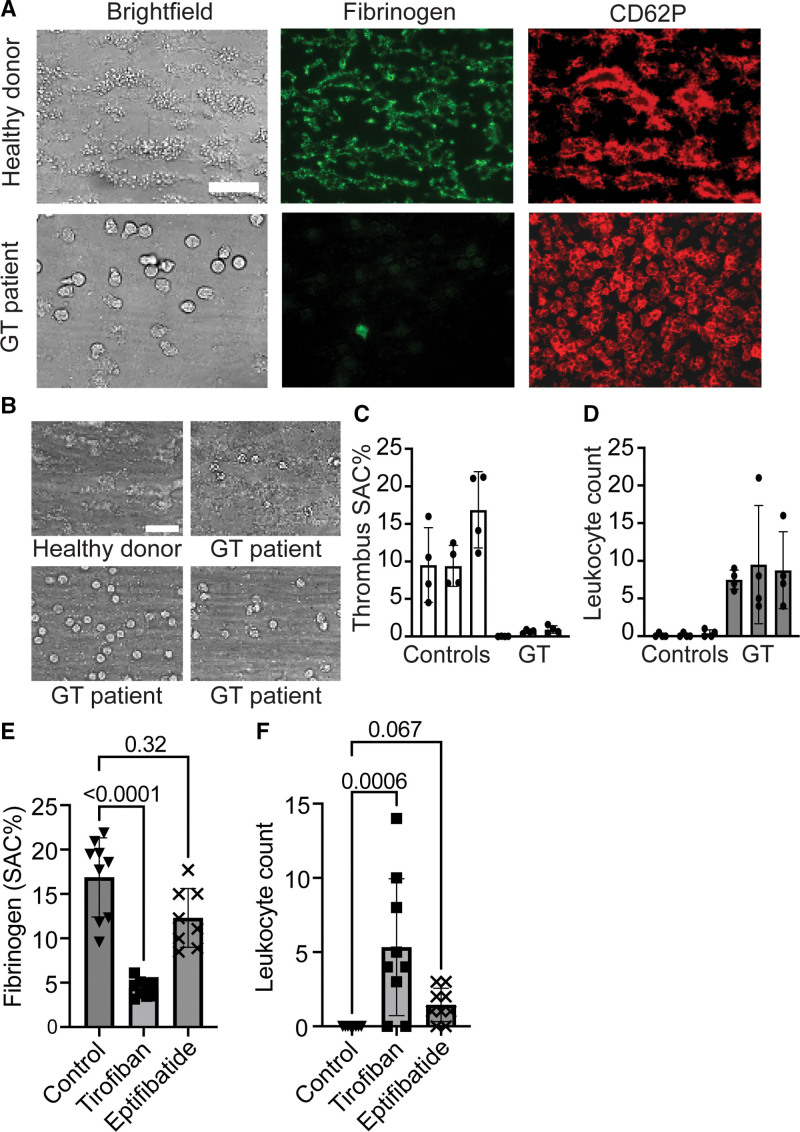
**Suppression of leukocyte adhesion to thrombi by integrin αIIbβ3 activation.** Recalcified whole blood from control subjects or patients with GT (Glanzmann thrombasthenia) was perfused over collagen at 1000 s^−1^ for 4 minutes. Thrombi were stained with fluorescein isothiocyanate (FITC)–labeled antifibrinogen monoclonal antibody (mAb) and Alexa Fluor 647 anti-CD62P mAb and observed by bright-field and multicolor fluorescence microscopy. **A**, Representative images of bright-field thrombi and bound fibrinogen (activated αIIbβ3) and CD62P (P-selectin) fluorescence. **B**, Bright-field images and quantified data of leukocyte count per field of 1.49 mm^2^. Scale bars, 25 µm. **C**, Surface area coverage (%SAC) of platelet adhesion. **D**, Leukocyte attachment. Counted were 4 microspots with blood from 3 day-control subjects (C1–C3) and 3 patients with Glanzmann thrombasthenia (P1–P3). Effect of tirofiban (1 µg/mL) or eptifibatide (10 µM) on platelet fibrinogen binding (**E**) and leukocyte adhesion (**F**) with blood from control subjects. Mean±SD (n=9), Kruskal-Wallis test with Dunn pairwise comparison test to control.

To examine the interactions of autologous leukocytes with activated platelets at arterial flow conditions, we perfused blood from control subjects and 3 unrelated patients with Glanzmann thrombasthenia (lacking platelet integrin αIIbβ3) at a wall shear rate of 1000 s^−1^ over a collagen surface, using a previously described microfluidics setup.^31^ Representative bright-field and fluorescence microscopic images after 4 minutes of flow with control blood showed the presence of large thrombi, with aggregated platelets showing high fibrinogen binding and CD62P expression (Figure 1A and 1B). A different picture was seen with the Glanzmann thrombasthenia blood samples, giving a monolayer of spread platelets essentially devoid of fibrinogen binding but with high CD62P expression and phosphatidylserine exposure (Figure S1).

When using the control blood samples, rolling or adhered leukocytes were hardly detected on the formed thrombi. Leukocyte numbers amounted to only 0.03±0.01 (mean±SD, n=10 subjects) per microscopic field (1.49 mm^2^). However, in the case of blood from the 3 patients with Glanzmann thrombasthenia, devoid of platelet integrin αIIbβ3 (Figure S2A), there was a profound increase up to 7.2±0.7 leukocytes per field (mean±SD, n=3), which was significantly higher than the number of adhered leukocytes using blood samples from healthy controls measured on the same day (Figure 1A through 1D). For all 3 patients, hematologic variables and counts of white and red blood cells and platelets were within normal ranges (Table S1).

These data suggested a suppressive effect of platelet integrin αIIbβ3 on the leukocyte adhesion to thrombi under flow. This suggestion was confirmed by the observation that αIIbβ3 blockage with the RGD mimetic tirofiban or the cyclic hArgGlyAsp hexapeptide eptifibatide (binding to different integrin epitopes)^33^ led to a substantial increase in leukocyte adhesion to collagen-attached platelets under flow (Figure 1E and 1F). Enforcement of integrin activation with 2 mmol/L MnCl_2_ did neither increase nor decrease the leukocyte attachment (not shown), which can be explained by studies that this cation did not support agonist-induced platelet aggregation.^34^

The finding that (collagen induced) activation of αIIbβ had a suppressive effect on leukocyte interaction was indirectly supported by flow-cytometric analysis of thrombin-activated blood from patients with Glanzmann thrombasthenia, indicating an increased formation of platelet-neutrophil conjugates when compared with control subjects (Figure S2A through S2C).

Based on observations that during blood flow leukocytes incidentally adhered at sites of flow disturbance downside of a thrombus, we decided to provoke a temporary flow disturbance by switching off the perfusion pump for 10 s, which led to a gradual slowing down of blood flow until stasis. This intervention resulted in a marked increase in adhered leukocytes (see next section) and, therefore, was used in further experiments.

### Adhesion of Neutrophils to Thrombi With Highly Activated Platelets

To determine whether leukocyte adhesion was dependent on the activation state of platelets in a thrombus, we generated thrombi by whole-blood perfusion over collagen spots (4 minutes, wall-shear rate of 1000 s^−1^). When using D-Phe-Pro-Arg chloromethyl ketone–anticoagulated blood, the thrombi formed with few phosphatidylserine-exposing platelets and were prone to disaggregate (Figure S3A and S3B); these were termed as type I thrombi.^22^ Coperfusion of the blood with ADP resulted in a more stabilized thrombus type (type II). By also initiating the coagulation process using coperfusion of recalcified blood with tissue factor,^23^ stable and contracted thrombi were formed, which were termed as type III. We observed that initial platelet deposition to the collagen was similar for all thrombus types, whereas the thrombus stability increased from type I to type III, as deduced from analysis of the surface area coverage of contracted thrombi (Figure S3B). Platelet phosphatidylserine exposure increased during incubation from type I to type II to III.

When monitoring the attachment of leukocytes by temporary flow disturbance after 4 minutes of blood perfusion, we noted a marked increase from type I to type II and III thrombi. By staining for CD15, we established that at all conditions >97% of the attached leukocytes were polymorphonuclear cells (Figure S3C). For the type II to III thrombi with activated platelets, the adhered cells stayed around the thrombus sites even on long-term incubation. As a control surface, we used immobilized fibrinogen, which gave a monolayer of platelets with low CD62P expression, not supporting leukocyte attachment (<0.05 per field of view).

Considering that the contracted type III thrombi contained highly activated platelets, these were used for further characterization of leukocyte interactions. In agreement with earlier data,^31^ we confirmed that the thrombi with activated platelet αIIbβ3 also bound fibrin(ogen) by making overlays of FITC PAC1 mAb and Alexa Fluor 647 fibrinogen images (Figure S4A). Multicolor confocal imaging indicated that the platelets in thrombi had undergone secretion of their α- and δ-granules, while a population of the platelets also showed phosphatidylserine exposure (Figure S4B). Multicolor staining indicated that the vast majority of the leukocytes on type III thrombi displayed CD11b and CD66b expression next to CD15 (Figure S4C). Furthermore, they expressed MPO and responded to fMLP (see below), identifying this majority as neutrophils.

Considering that platelet activation can induce NET formation of neutrophils,^8^ we examined this process for the cells adhered to type I to III thrombi. Interestingly, at an early time point and after 2 to 16 hours of incubation, no more than few cells with a distorted nucleus could be observed (Figure S5A). To confirm the scarcity of NET-forming cells on platelets, the leukocytes attached to type III thrombi were postincubated with the protein kinase C activator PMA or the bacterial endotoxin lipopolysaccharide, both of which as established inducers of NET formation.^35^ These treatments profoundly increased the number of adhesive cells with a distorted nucleus (4′,6-diamidino-2-phenylindole), staining highly for MPO and citrullinated histone 3, indicative of the excretion of long DNA filaments (Figure S5B and S5C). Together, these results pointed to stasis-enforced, continued neutrophils interacting with the thrombi of activated platelets.

### Platelet CD40L Controlling the Movement Pattern of Neutrophils on Thrombi

**Figure 2.**
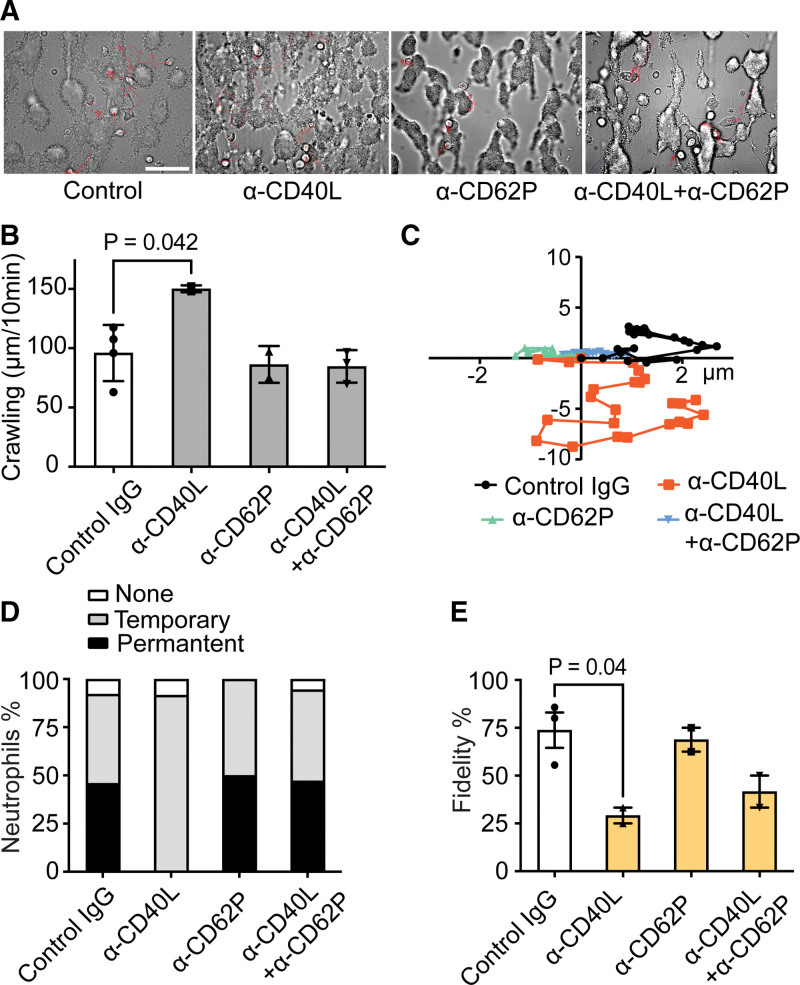
**Role of the platelet-expressed adhesive receptor CD40L in leukocyte movement at thrombi.** Type III thrombi with leukocytes were generated (Figure S3), and movements of the cells were recorded as bright-field movies for 10 minutes. Blocking with anti-CD62P (P-selectin) or anti-CD40L monoclonal antibody (mAb), or control mAb (each 10 µg/mL), as indicated. **A**, Bright-field images with movement patterns of several thrombus-adhered cells (red traces). Bar, 50 µm. **B**, Average crawling velocity of moving cells per condition. **C**, Movement directionality of representative cells per condition over time. **D**, Analysis of leukocyte microlocation with temporary or permanent location at a thrombus, and of cells with single-thrombus fidelity (**E**) during 10 minutes. Mean±SE (n=10–15 cells), Kruskal-Wallis test.

Given the ability of leukocytes to interact with surface receptors present on (activated) platelets, such as integrins, CD62P, and CD40L,^14,36^ we studied how these receptors contributed to the adhesion to type III thrombi. Therefore, videos from the thrombi (10 minutes) were analyzed for cellular movements. Analysis of single-cell movement patterns showed a mean crawling velocity of 90 µm/h of single leukocytes, regularly visiting an adjacent thrombi (Figure 2A through 2C). Antibody blockage of platelet-expressed CD40L did not reduce the number of cells per field but increased their overall movement velocity, hence suggesting a weakened interaction with the thrombi. Interestingly, such an increased crawling rate was not observed on blockage of platelet CD62P (Figure 2B and 2C). As a measure of the chemotactic activity of thrombi, we also analyzed videos for the fidelity of individual cells to stay at or around a given thrombus. The blockage of CD40L, but not of CD62P, significantly reduced the fraction of cells staying attached to any thrombus (Figure 2D), which we interpreted as a high thrombus fidelity (Figure 2E). The movement patterns were also confirmed by time-dependent distance profile analyses (Figure S6). Jointly, these data pointed to a more important role of platelet CD40L than of CD62P in the time-dependent microlocation of the cells, in majority neutrophils.

### Platelet-Released Substances From Thrombi Inducing Neutrophil Activation

To further examine the intercellular communication, we purified granulocytes from whole blood and loaded the collected cells with the cytosolic Ca^2+^ probe Fluo-4-AM. Markedly, the combined addition of platelets and thrombin (4 nmol/L)—but not of platelets or thrombin alone—induced a high [Ca^2+^]_i_ rise in the Fluo-4–loaded cells (Figure S7A and S7B). As a control condition, we used the neutrophil-specific G-protein–coupled receptor agonist, fMLP (1 µmol/L).^37^ This agonist-induced a strong Ca^2+^ response in essentially all fluorescent cells (Figure S7C). The platelet inhibitor iloprost (10 nmol/L), evoking cyclic AMP elevation in platelets, had no effect on the granulocyte Ca^2+^ response (not shown). In confirmation with the neutrophil responses to fMLP, in flow cytometry with FITC anti-CD66b mAb we observed an fMLP-induced fluorescence increase from 15.2±1.6 MFI to 32.7±6.6 MFI (mean±SD, n=3).

**Figure 3.**
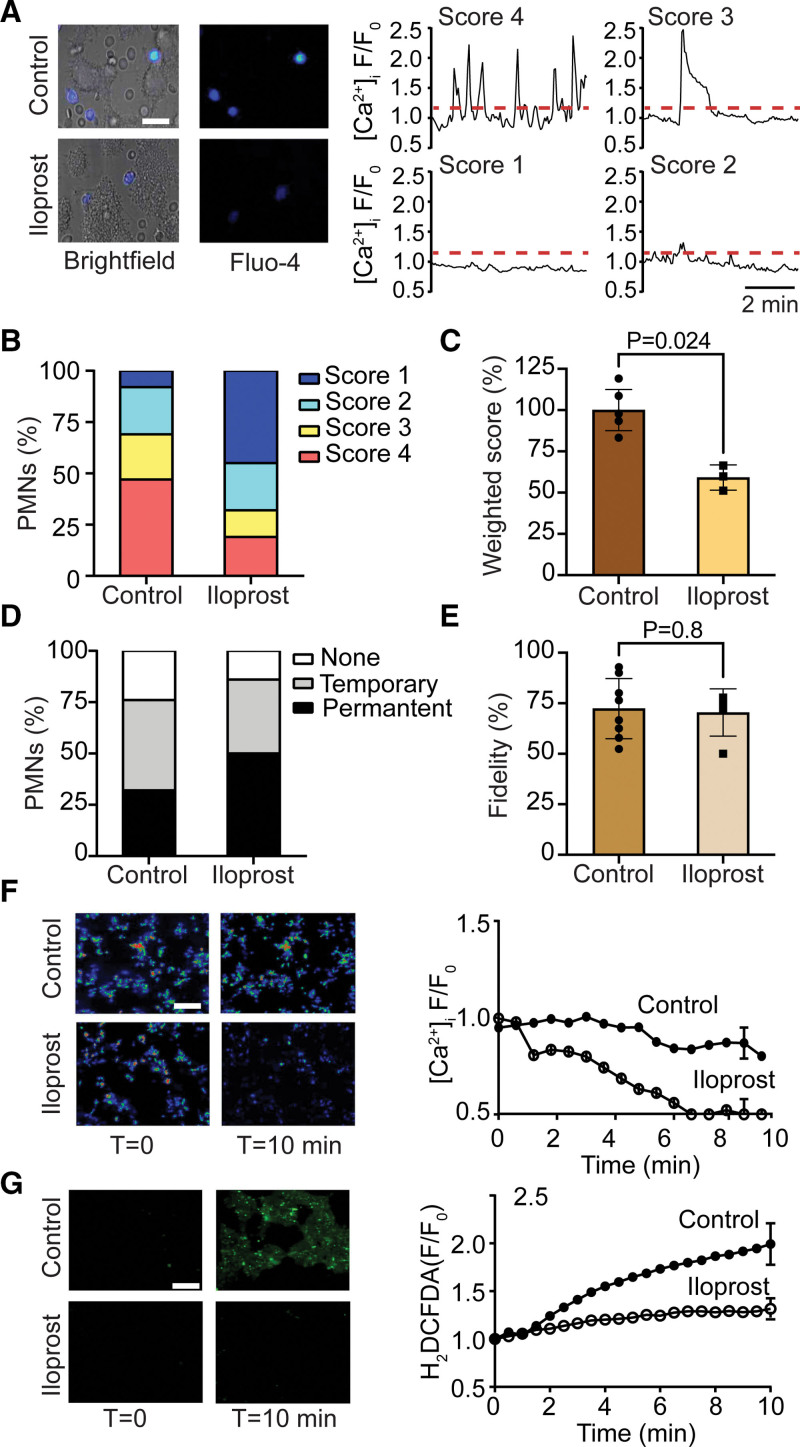
**Inhibited leukocyte activation by thrombus treatment with iloprost.** Type III thrombi containing leukocytes were left untreated (control) or postperfused with iloprost (10 nmol/L) for 10 minutes, and activation markers of leukocytes (neutrophils) or platelets were measured. **A** through **C**, Effect of iloprost (*t*=0) on rises in [Ca^2+^]_i_ of Fluo-4–loaded cells adhered to thrombi. Recorded were 10-minute fluorescence and bright-field movies. **A**, Representative images at 10 minutes after iloprost, and [Ca^2+^]_i_ transients of single cells with high or lower Ca^2+^ scores. Bars, 25 µm. Scores 1–4 were defined on the amplitude and time fraction of signal above resting level (see methods), which was set as *F/F*_*o*_ 1.15 (dotted lines). Distribution profiles of Ca^2+^ scores (**B**) and calculated weighted Ca^2+^ scores (**C**) of >50 polymorphonuclear cells (PMN) per condition. **D** and **E**, Effect of iloprost on leukocyte microlocation, temporary or permanent at a thrombus (**D**), and on cell fidelity to a thrombus (**E**). **F**, Effect of iloprost on [Ca^2+^]_i_ rises in Fluo-4–loaded platelets in thrombi. Representative images and overall changes in *F/F*_*o*_ (*F*_*o*_=basal fluorescence *t*=0). **G**, Effect of iloprost on the production of reactive oxygen species (ROS), measured from H_2_DCFDAR (the ROS-sensitive probe) fluorescence in postlabeled thrombi. Shown are representative images and rise in ROS production. Note near-complete silencing of thrombi after iloprost treatment. Mean±SE (n=3–5), Mann-Whitney *U* test.

Subsequently, we supplemented the Fluo-4–loaded granulocytes (in majority neutrophils) with autologous blood samples and perfused these over type III thrombi while recording bright-field and fluorescence video images. Video analysis of pseudoratioed [Ca^2+^]_i_ traces revealed a marked heterogeneity between the adhered cells that were crawling on and around thrombi (Figure 3A; Video S1). About 50% of the cells displayed repetitive, high amplitude [Ca^2+^]_i_ transients, categorized as Ca^2+^ score 4 (Figure 3B). A quarter of the cells showed a single, high amplitude (*F/F*_*o*_>1.5) rise in [Ca^2+^]_i_ (ie, Ca^2+^ score 3), and another quarter had continuous low-amplitude [Ca^2+^]_i_ transients (ie, Ca^2+^ score 2). Less than 10% of the cells were considered essentially silent with no marked increase of [Ca^2+^]_i_ (*F/F*_*o*_<1.1, that is, Ca^2+^ score 1).

Immune staining at these conditions confirmed that the majority of adhered cells were neutrophils (not shown), in agreement with the high responsiveness to fMLP. To find out whether the prolonged Ca^2+^ signaling relied on the production of platelet-derived mediators, we posttreated the thrombi with iloprost (10 nmol/L), which acutely changed the Ca^2+^ response patterns in the adhered cells (Video S2). After iloprost postperfusion, only ≈20% of the Fluo-4–loaded cells were scored 4, whereas over 40% of the cells scored 1 (Figure 3A and 3B). The reduced responsiveness was also reflected by a lower weighted Ca^2+^ score (Figure 3C). Interestingly, iloprost treatment also increased the cells that stayed at a given thrombus, although their (high) fidelity was unchanged (Figure 3D and 3E).

These findings suggested a long-term high activation and secretion of platelets in the thrombi, being reverted by cAMP elevation with iloprost. To confirm the iloprost effect, we generated type III thrombi containing 20% autologous Fluo-4–loaded platelets. Imaging analysis now indicated that the labeled platelets had an overall high Ca^2+^ signal, which stayed high during a 10-minute interval (Figure 3F). In contrast, on iloprost treatment, the Fluo-4 fluorescence started to decline, until a basal level of fluorescence was reached. In addition, iloprost treatment suppressed the production of reactive oxygen species by activated platelets, as detected with the probe H_2_DCFADA (Figure 3G). Together, these findings indicate that iloprost suppressed the activation of platelets in a thrombus, with as a consequence diminished [Ca^2+^]_i_ transients in adhered neutrophils.

### Platelet-Released Chemokines Triggering Neutrophil Activation

**Figure 4.**
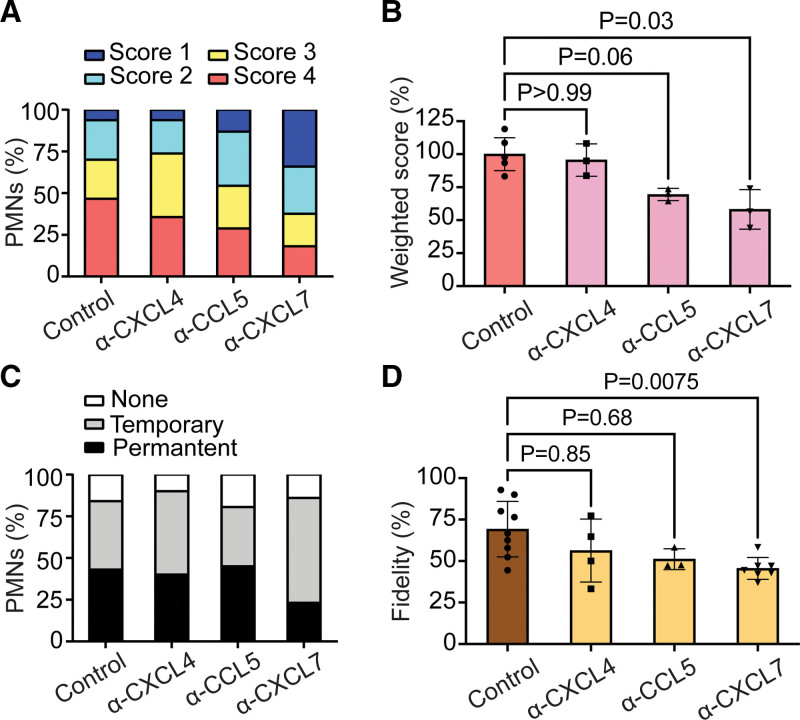
**Regulation of leukocyte Ca^2+^ rises and location by platelet-released chemokines.** Type III thrombi were perfused for 2 minutes with control IgG (vehicle) or blocking antibody against CXCL4, CCL5, or CXCL7 (1 µg/mL) and postperfused with autologous Fluo-4–loaded polymorphonuclear cells (PMNs). Transient rises in [Ca^2+^]_i_ and chemotactic responses in leukocytes were evaluated for 10 minutes by bright-field and fluorescence imaging (see Figure 3). **A** and **B**, Effect of blocking antibodies on distribution profile of Ca^2+^ scores (**A**) and on weighted Ca^2+^ score (**B**) of >50 neutrophils from 3 to 4 independent experiments. **C** and **D**, Effect of blocking antibodies on preferred cell location with temporary or permanent location at a thrombus (**C**), and of cells with single-thrombus fidelity (**D**). Mean±SE (n=3–4). *P* values relative to control condition (Kruskal-Wallis test with Dunn pairwise comparison test to control for **B** and **D**, or χ^2^ test for **A** and **C**).

Considering that platelet-released chemokines can trigger neutrophils,^38,39^ we studied this for the platelets in type III thrombi. We first confirmed the presence of secreted CCL5 (RANTES) and CXCL7 (NAP2) on thrombi by immunostaining (Figure S8). Using blocking antibodies, we examined the effects on the leukocyte activation responses. Analysis of the changes in Fluo-4 fluorescence indicated that the blockage of CCL5 or CXCL7, but not of CXCL4 resulted in lower Ca^2+^ signals (Figure 4A and 4B). For CXCL7 blockage, this resulted in a significant reduction in cells with a permanent location and hence lower thrombus fidelity (Figure 4C and 4D). Regarding CCL5 blockage, the location effect was not significant.

To confirm these findings, we stimulated purified, Fluo-4–loaded granulocytes with CXCL7 or CCL5 in static conditions. Both chemokines induced strong but distinct [Ca^2+^]_i_ rises. Exposure of the cells to CXCL7 resulted in a synchronous single, large transient rise in [Ca^2+^]_i_, which halted the movement pattern (Figure S9A through S9D). However, exposure to CCL5 led to a [Ca^2+^]_i_ spiking pattern, which in this case was accompanied by crawling during the [Ca^2+^]_i_ rises (Figure S9E through S9H). In addition, we perfused the Fluo-4–loaded cells with the releasate from activated platelets. This also resulted in repetitive [Ca^2+^]_i_ spiking of relatively high amplitude, accompanied by cell movements in between the spikes (Figure S9I through S9L). Also, in this condition, a consistent high responsiveness to fMLP confirmed the neutrophil nature of most of the cells with high CD66b expression (not shown). Taken together, these data point to a combined effect of platelet-released chemokines—including CCL5 and CXCL7—on neutrophil signaling responses.

### Dual Role of Integrin αIIbβ3 in Neutrophil Adhesion and Activation

**Figure 5.**
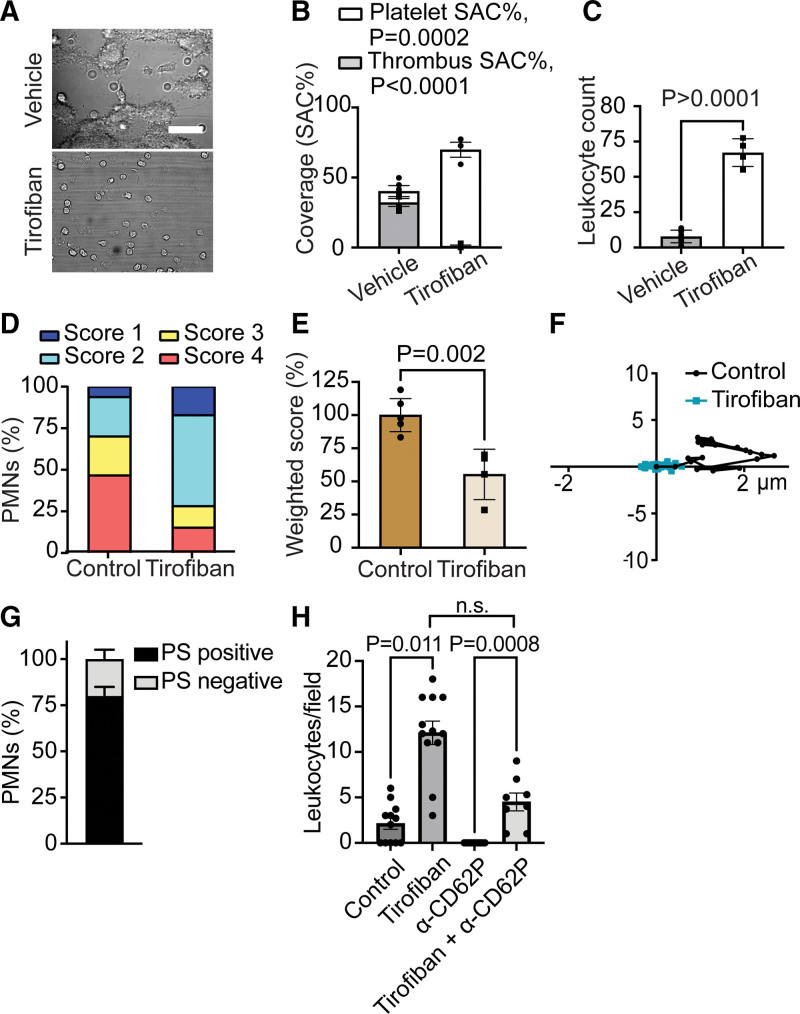
**Suppression of leukocyte adhesion and activation by αIIbβ3 inhibition.** Whole-blood containing vehicle medium or tirofiban (1 µg/mL) was flowed over collagen to generate type III thrombi and adhesion and activation of (Fluo-4–loaded) leukocytes were monitored. **A**, Representative bright-field images after flow showing increased cell attachment in the presence of tirofiban; bar, 25 µm. **B**, Quantification of all deposited platelets and multilayered thrombi. **C**, Leukocyte count per field. **D**, Distribution profile of Fluo-4–loaded neutrophils according to Ca^2+^ scores. **E**, Overall weighted Ca^2+^ score. Mean±SE (n=3), Mann-Whitney *U* test **F**, Directionality of movements of representative cells (10 minutes) with vehicle or tirofiban (1 µg/mL), as for Figure 2C. **G**, Distribution profile of polymorphonuclear cells (PMNs) over time interacting with phosphatidylserine (PS)-exposing platelets, as detected with fluorescein isothiocyanate (FITC)–annexin A5. **H**, Single or combined effect of added tirofiban (1 µg/mL) and blocking anti-CD62P monoclonal antibody (mAb; 10 µg/mL). Mean±SE (n=8–12), Kruskal-Wallis test followed by Dunn multiple comparison test. SAC indicates surface area coverage.

Taking further the experiments with Glanzmann blood, we examined the effects of integrin αIIbβ3 blockage with tirofiban on the [Ca^2+^]_i_ transients of leukocytes. Although tirofiban treatment prevented the platelet aggregate formation, resulting in a monolayer of platelets (Figure 5A through 5C), the multiple adhered leukocytes had a low Ca^2+^ score (Figure 5D and 5E) and a minimal movement pattern (Figure 5F). This was confirmed in Rose plots and traveling distance profiles (Figure S10). Interestingly, in this condition, a relatively large fraction of the cells were attaching to phosphatidylserine-exposing platelets (Figure 5G), which could be confirmed by multicolor fluorescence imaging (Figure S11) and recorded videos (Video S3). In addition, we found that the tirofiban-induced leukocyte attachment was nonsignificantly reduced up to 62% on the blockage of platelet CD62P (Figure 5H).

**Figure 6.**
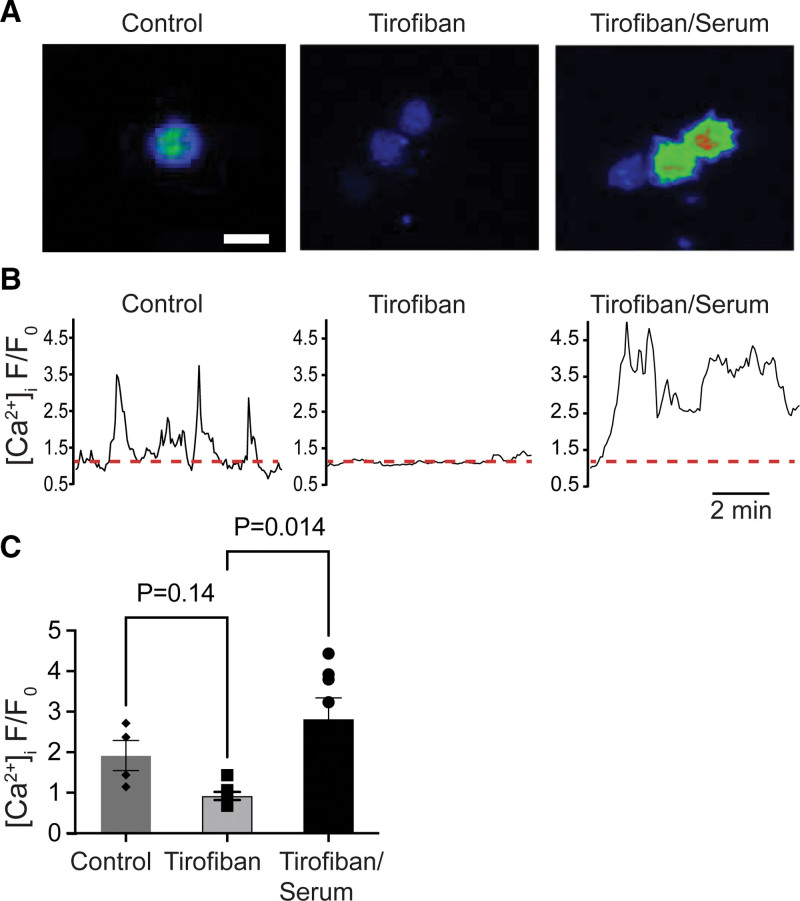
**Serum-induced activation of leukocytes at thrombi.** Type III thrombi with adhered Fluo-4–loaded leukocytes, formed under control conditions or in the presence of tirofiban (1 µg/mL), were postperfused with vehicle medium or autologous serum (10 minutes, 200 s^−1^). Shown are (**A**) representative fluorescence images (bar, 25 µm); (**B**) Ca^2+^ transients of a representative cell; and (**C**) quantified rises in [Ca^2+^]_i_. Mean±SD (n=4–7 cells from 2 independent experiments), Kruskal-Wallis test followed by Dunn multiple comparison test to tirofiban treatment.

To check for the ability of platelet release substances on the Ca^2+^ responses, we postperfused the cells with freshly prepared autologous serum—enriched in platelet secretion products—which greatly increased the [Ca^2+^]_i_ transients of the leukocytes (Figure 6A through 6C). This postperfusion also induced attachment and activation/spreading of cells in a similar manner as did fMLP (Figure S12). Jointly, our results suggest a dual effect of activated platelet αIIbβ3, by on one side preventing adhesion of the in majority neutrophils and on the other side suppressing the platelet release of neutrophil-stimulating substances.

## DISCUSSION

In this article, using microfluidics approaches, we identified several mechanisms regulating the interactions of human leukocytes—in majority neutrophils—with platelets in a thrombus under flow conditions: (1) A to date undisclosed role of activated platelet integrin αIIbβ3 suppressing leukocyte adhesion, an effect that could be overcome by short-term flow disturbance. (2) A role of platelet-expressed CD40L in controlling the movement pattern and fidelity of leukocytes adhered to a thrombus. (3) An activation but not adhesion-stimulating effect on the cells by platelet secretion substances, which continued until inhibition of the platelets with iloprost. (4) A likely role of platelet-derived chemokines on the leukocytes in the order of CXCL7>CCL5>CXCL4. (5) A no more than limited NET formation despite activation of the identified neutrophils, such in contrast to posttreatment of the cells with PMA or lipopolysaccharide. Together, these findings reveal an unexpected, multifaceted pattern of leukocyte (neutrophil)-platelet interactions during and after thrombus formation, involving the functional activities of these cell types.

Neutrophils, as active secretory cells, are known to respond to receptor agonists via CXCR2 and other G-protein–coupled receptors by way of [Ca^2+^]_i_ rises, degranulation, and migration.^40,41^ Here, we observed spiking [Ca^2+^]_i_ transients in such cells evoked by applied chemokines in the order of CXCL7>CCL5>>CXCL4. Because we also observed that some of the chemokines were attached to thrombi, and blocking antibodies impacted the leukocyte movements, there may be a role for chemokines in the adhesion. Indeed, activated platelets are known to present chemokines that recruit other blood cells.^42^ In support of our findings, other authors have shown that the chemokine CXCL7, acting via CXCR1/2, promotes in vivo leukocyte migration into a platelet thrombus at sites of vascular injury.^43^ Confirming a role of platelet-derived CCL5 is the observation that this chemokine regulates neutrophil activation in acute experimental colitis.^44^ While our findings point to nonredundant roles of platelet-released CXCL7 and CCL5 in the activation of in majority adhered neutrophils, there likely are additive roles of other platelet substances, such as platelet-activating factor,^45^ TREM-1 ligand,^46^ and cyclooxygenase or lipoxygenase products.^47,48^

A new result was the observed inhibitory role of activated integrin αIIbβ3 on platelets in suppressing the attachment of leukocytes under flow conditions. In line with this, also flow-cytometric observations under stasis indicated that the formation of platelet-neutrophil conjugates after thrombin stimulation is increased in patients with Glanzmann thrombasthenia (Figure S2). Platelets from these patients with a bleeding phenotype,^25^ lacking integrin αIIbβ3, are reported on activation to display normal expression levels of CD62P and CD40L.^49,50^ Although the inhibitory counter-receptor or mechanism for αIIbβ3 on neutrophils is unknown, we propose that this integrin functions in concert with known positive interactions between activated platelets and neutrophils, such as the axes ICAM2-αLβ2 and glycoprotein Ib-αMβ2, for instance in the context of thromboinflammation.^51,52^

Whereas posttreatment of the adhered neutrophils with PMA or lipopolysaccharide provoked NETosis, as observed by nuclear distortion, the mere interaction of the cells with a thrombus did no more than limitedly cause this response. Apparently, the neutrophil stimulation by platelet substances, leading to [Ca^2+^]_i_ transients, is a too weak or a too short protein kinase C stimulus for inducing the NET formation. A large body of research points to the role of NETs in inflammatory and thrombotic diseases,^9,53^ for instance, promoted by platelet-expressed CD62P^35^ or chemokines.^15^ However, our in vitro findings suggest that this process is not an initial event in thrombus formation.

In seeming contrast to our findings, other authors reported an increased neutrophil and T-cell adhesion of preactivated platelets via SLC44A2 (solute carrier family 44 member 2) to αIIbβ3, resulting in NET formation in a time frame of 2 hours.^54^ We speculate that those data concern more severe neutrophil-activating conditions in the microchannels used, which may have overcome an inhibitory effect of αIIbβ3 on flow-dependent neutrophil adhesion.

A limitation of our study was the application of momentary flow interruption to obtain a consistently high adhesion of leukocytes in the absence of integrin blockage. Yet, flow disturbances are well known—and have been modeled in whole-blood studies—under conditions of venous and arterial thrombosis.^30^ Given the thrombogenic consequences of neutrophil-thrombus interactions,^7^ pharmacological intervention in these interactions is becoming interesting. The here presented microfluidic approach to simultaneously determine adhesion, movement, and activation responses of neutrophil-enriched leukocytes may, therefore, present a powerful tool for further investigation of thromboinflammatory disease states and possible pharmacological interventions.

## ARTICLE INFORMATION

### Acknowledgments

C. Schönichen and M. Nagy performed experiments, analyzed and interpreted data, and wrote the article. S.L.N. Brouns and S.J. Montague performed experiments and analyzed data. R.R. Koenen provided essential materials, contributed with ideas, and revised the article. J.J. Burston and V.B. O’Donnell contributed with ideas and revised the article. F. Ní Áinle provided essential materials. K. Jurk and B.E. Kehrel recruited patients, performed experiments, and revised the article. O. Soehnlein designed research, supervised research, and interpreted data. J.M.E.M. Cosemans designed research, supervised research, interpreted data, and revised the article. S.P. Watson contributed with ideas, supervised research, interpreted data, and revised the article. M.J.E. Kuijpers supervised research and revised the article. J.W.M. Heemskerk designed and coordinated the research and wrote the article.

### Sources of Funding

This work was supported by the Cardiovascular Center (HVC), Maastricht University Medical Center^+^; the Interreg program Euregio Meuse-Rhin (Polyvalve); the European Union’s Horizon 2020 research and innovation program under the Marie Skłodowska-Curie grant agreement 813409. C. Schönichen is enrolled in a joint PhD program of the Universities of Maastricht and Mainz. S.J. Montague is supported by the British Heart Foundation, AA/18/2/34218. J.M.E.M. Cosemans is supported by the Netherlands Organization for Scientific Research (NWO; Vidi 91716421).

### Disclosures

J.W.M. Heemskerk is scientific advisor of the Synapse Research Institute Maastricht. The other authors report no conflicts.

### Supplemental Material

Supplemental Methods

Figures S1–S12

Videos S1–S3

Table S1

Major Resources Table

Supplemental References 1–3

## Supplementary Material


