# Supplementary material for: Antagonistic Roles of Human Platelet Integrin αIIbβ3 and Chemokines in Regulating Neutrophil Activation and Fate on Arterial Thrombi Under Flow
Source: Arterioscler Thromb Vasc Biol. 2023 Jul 6;43(9):1700–12. doi: 10.1161/ATVBAHA.122.318767 (PMC10443630; doi:10.1161/ATVBAHA.122.318767)
Supplement: Supplementary file 1 [file atv-43-1700-s001.pdf]

SUPPLEMENTAL MATERIALS *ATVB/2011/317230 revised*

Antagonistic Roles of Human Platelet Integrin αIIbβ3 and Chemokines in Regulating Neutrophil Activation and Fate on Arterial Thrombi under Flow

Claudia Schönichen[1,2], Samantha J. Montague[3], Sanne L.N. Brouns[1], James J. Burston[4], Judith M.E.M. Cosemans[1], Kerstin Jurk[2,5], Beate E. Kehrel,[5] Rory R. Koenen[1], Fionnuala Ní Áinle[6,7], Valerie B. O'Donnell[4], Oliver Soehnlein[8,9,10], Steve P. Watson[1,3,11], Marijke J.E. Kuijpers[1,12], Johan W.M. Heemskerk[1,13]*, Magdolna Nagy[1]*

[1]Dept. of Biochemistry, Cardiovascular Research Institute Maastricht (CARIM), Maastricht University, P.O. Box 616, 6200 MD, Maastricht, the Netherlands.
[2]Center for Thrombosis and Hemostasis, University Medical Center of the Johannes Gutenberg-University of Mainz, Langenbeckstr. 1, 55131 Mainz, Germany.
[3]Institute of Cardiovascular Sciences, The Medical School, University of Birmingham, Birmingham B15 2TT, United Kingdom.
[4]Systems Immunity Research Institute, School of Medicine, Cardiff University, CF14 4XN, United Kingdom.
[5]Dept. of Anaesthesiology and Intensive Care, University Hospital Muenster, Germany
[6]School of Medicine, University College Dublin, Dublin, Ireland.
[7]Dept. of Haematology, Mater Misericordiae University Hospital and Rotunda Hospital, Dublin, Ireland.
[8]Institute for Cardiovascular Prevention, Ludwig-Maximilians-Universität München, Munich, Germany.
[9]Institute for Experimental Pathology, Center for Molecular Biology of Inflammation, Westfälische Wilhelms Universität, Münster, Germany.
[10]Dept. of Physiology and Pharmacology, Karolinska Institutet, Stockholm, Sweden.
[11]Centre of Membrane Proteins and Receptors (COMPARE), Universities of Birmingham and Nottingham, the Midlands, United Kingdom.
[12]Thrombosis Expertise Centre, Heart and Vascular Centre, Maastricht University Medical Centre[+], Maastricht, the Netherlands.
[13]Synapse Research Institute, Kon. Emmaplein, 6217 KD, Maastricht, the Netherlands.

*These authors equally contributed to this work.

Correspondence to: Marijke J.E. Kuijpers, PhD (marijke.kuijpers@maastrichtuniversity.nl), Johan W.M. Heemskerk, PhD (jwmheem722@outlook.com), and Magdolna Nagy, PhD (m.nagy@maastrichtuniversity.nl), Department of Biochemistry, Maastricht University, P.O. Box 616, 6200 MD Maastricht, the Netherlands

Running head: Human Platelets and Neutrophils under Flow

EXTENDED MATERIALS AND METHODS

A **Major Resources Table** is included at the end of this document.

Blood Cell Preparation

Where indicated, polymorphonuclear leukocytes were enriched from heparinized blood using a histopaque-1077 and histopaque-1119 density gradient centrifugation method, essentially as indicated.[1] Briefly, a mixture of equal volumes of heparinized blood, histopaque-1077 and histopaque-1119 was centrifuged at 700*g* for 30 minutes without brake. The granulocyte-containing layer was removed, then washed twice with 10 mL isotonic phosphate-buffered saline, and recentrifuged at 350*g* for 10 minutes. The pelleted granulocytes, containing >90% neutrophils, were resuspended at a concentration of 1×10^6 cells/mL into Hepes buffer pH 7.45 (10 mmol/L Hepes, 136 mmol/L NaCl, 2.7 mmol/L KCl, 2 mmol/L $MgCl_2$, 0.1% glucose and 0.1% bovine serum albumin). Cell suspensions were supplemented with 2 mmol/L $CaCl_2$ before use. Purity and density of the cell suspensions were assessed with a Sysmex-XP300 hematology analyzer.

Flow Cytometry

Platelet integrin αIIbβ3 expression levels (based on PE anti-CD41 mAb staining), and platelet-neutrophil conjugates in diluted, thrombin-stimulated whole blood (based on PerCP anti-CD42a mAb staining) were assessed, as described before.[2]

Purified suspensions of granulocytes were evaluated for activation by flow cytometry, using an Accuri C6 flow cytometer and C-Flow Plus software (Becton-Dickinson Bioscience). Neutrophils were gated in forward/side scatter plots from the positive signal with FITC anti-CD66b mAb (1 µg/mL). Activation was assessed from cells containing elevated CD66b expression, MPO expression and/or ROS staining using CellRox Deep Red. After gating, mean fluorescence intensities of events were recorded. Where indicated, the granulocytes were pre-stimulated with N-formyl-methionine leucyl phenylalanine (fMLP, 1-4 µmol/L) or CCL5 (500 nmol/L).

Parameter analysis of thrombus formation

Brightfield and fluorescence images, recorded from thrombi formed after whole-blood perfusion flow over indicated collagen surfaces, were analyzed by observers blinded to the condition using previously described scripts in Fiji/ImageJ (supplement of Ref. [3]). In brief, surface area coverage % per image type was obtained by background correction, object recognition, and semi-automated (operator-dependent) intensity threshold settings. Thrombus morphological scores were compared to reference images and set as: 0, no adhesion; 1, single platelets; 2, platelet monolayer; 3, platelet aggregates; 4, large aggregates; and 5, end-stage aggregates.

Analysis of Leukocyte Ca^{2+} Rises and Movement Patterns

For defined trace analyses of single-cell rises in $[Ca^{2+}]_i$, the confocal time stacks were imported in Matlab with the OME Bio-Formats toolbox (https://www.openmicroscopy.org/), and then processed using DIPImage (http://www.diplib.org/). In short, the stacks were filtered by a 3D median filter and thresholded to a fixed value. Objects (cells) touching the image-border anytime during the acquisition, and objects that split into multiple objects were rejected from the binary mask. The 3D objects were then assigned in time frames of individual cells, where for each time frame the intensity was determined. The single cell fluorescence traces were normalized to the resting level (F/F_o), and analyzed for responsiveness using GraphPad Prism software.

Confocal time series were also analyzed for movement patterns of individual cells, using Fiji software. Movement profiles were classified as: <20% contact with platelet thrombi, a temporary 20-80% contact, or a persistent >80% contact with the thrombi. Regarding directionality of the movement, contacting cells were classified as: low fidelity (switching between thrombi) or high fidelity (>80% stay on or return to same thrombus).

Additional References

1. Kulkarni S, Woollard KJ, Thomas S, Oxley D, Jackson SP. Conversion of platelets from a proaggregatory to a proinflammatory adhesive phenotype: role of PAF in spatially regulating neutrophil adhesion and spreading. *Blood*. 2007; 110: 1879-1886.

2. Jurk K, Ritter MA, Schriek C, Van Aken H, Droste DW, Ringelstein EB, Kehrel BE. Activated monocytes capture platelets for heterotypic association in patients with severe carotid artery stenosis. *Thromb Haemost*. 2010; 103: 1193-1202.
3. Huang J, Jooss NJ, Fernandez DI, Sickmann A, Garcia A, Wichapong K, Dijkgraaf I, Heemskerk JWM. Roles of focal adhesion kinase PTK2 and integrin αIIbβ3 signaling in collagen- and GPVI-dependent thrombus formation under shear. *Int J Mol Sci*. 2022; 23: 8688.

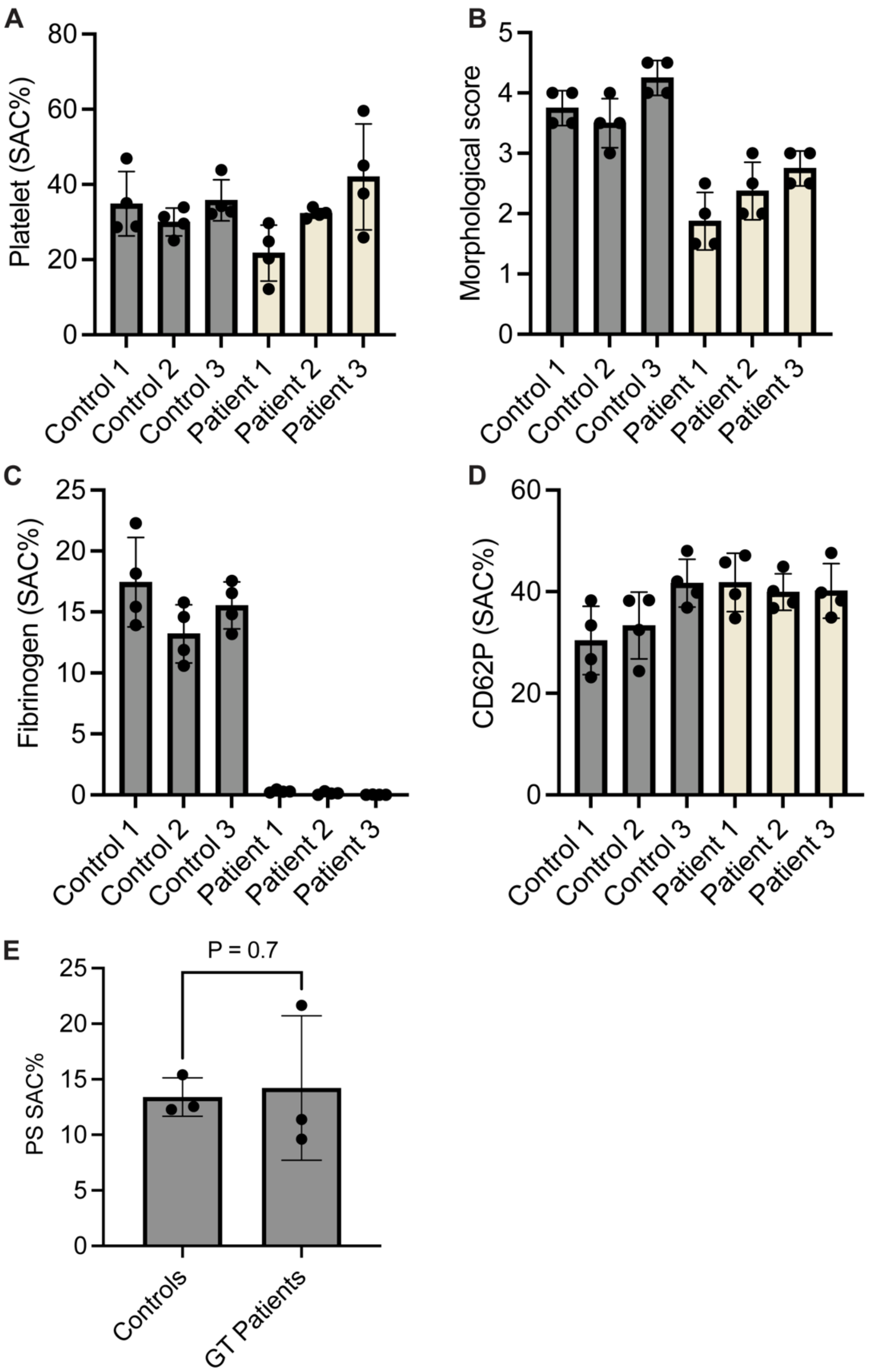

Figure S1. Abrogated platelet aggregation and fibrinogen binding upon collagen-induced thrombus formation with blood from patients with Glanzmann's thrombasthenia (GT). Recalcified whole blood from control subjects or Glanzmann thrombasthenia patients was perfused over collagen at 1000 s^{-1} during 4 minutes. Thrombi were stained with FITC anti-fibrinogen mAb and AF647 anti-CD62P mAb and analyzed for SAC%, as for Figure 1. Shown are per subject parameter values of platelet adhesion (**A**), thrombus morphological score, scaled 0-5 as in materials (**B**); and furthermore, after staining and rinse: fibrinogen binding (**C**), and CD62P expression (**D**). Note low thrombus morphology and absence of fibrinogen binding for all three Glanzmann patients. Mean±SE (n=4).

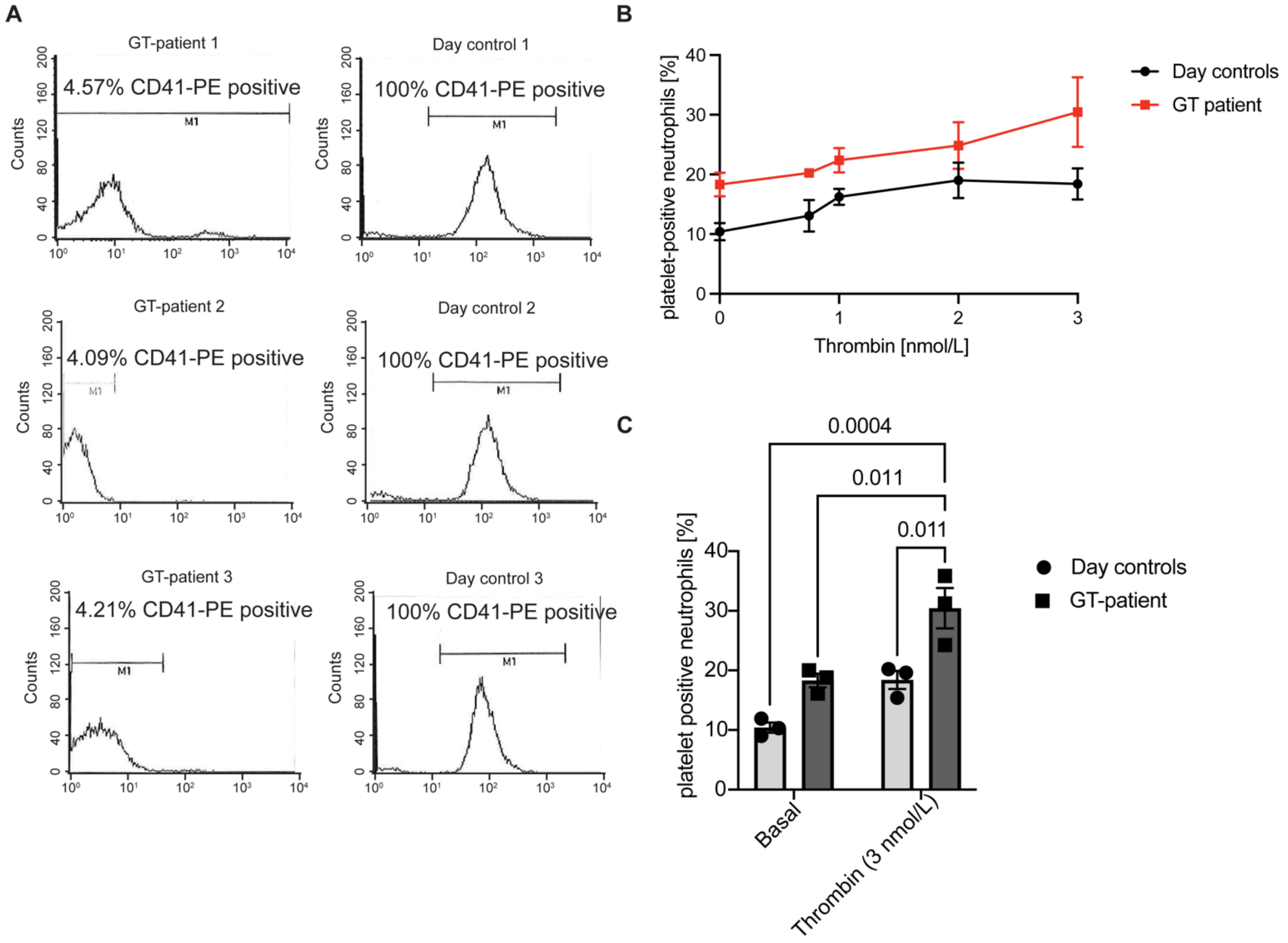

Figure S2. Increased platelet-neutrophil interaction in activated blood from patients with Glanzmann's thrombasthenia (GT). A, Flow cytometric histograms of PE-labeled anti-CD41 mAb platelets from Glanzmann patient and indicated day-control subject. **B**, **C**, Flow cytometric analysis of platelet-neutrophil conjugates in diluted blood from day controls and a GT patient (three different samples) upon stimulation with thrombin in the presence of fibrin polymerization inhibiting peptide GPRP. Neutrophils were gated from typical side scatter characteristics, and platelet-bound neutrophils were identified from staining by PerCP anti-CD42a mAb. Percentages of platelet-neutrophil conjugates were quantified. Mean±SD (n=3), 2-way ANOVA.

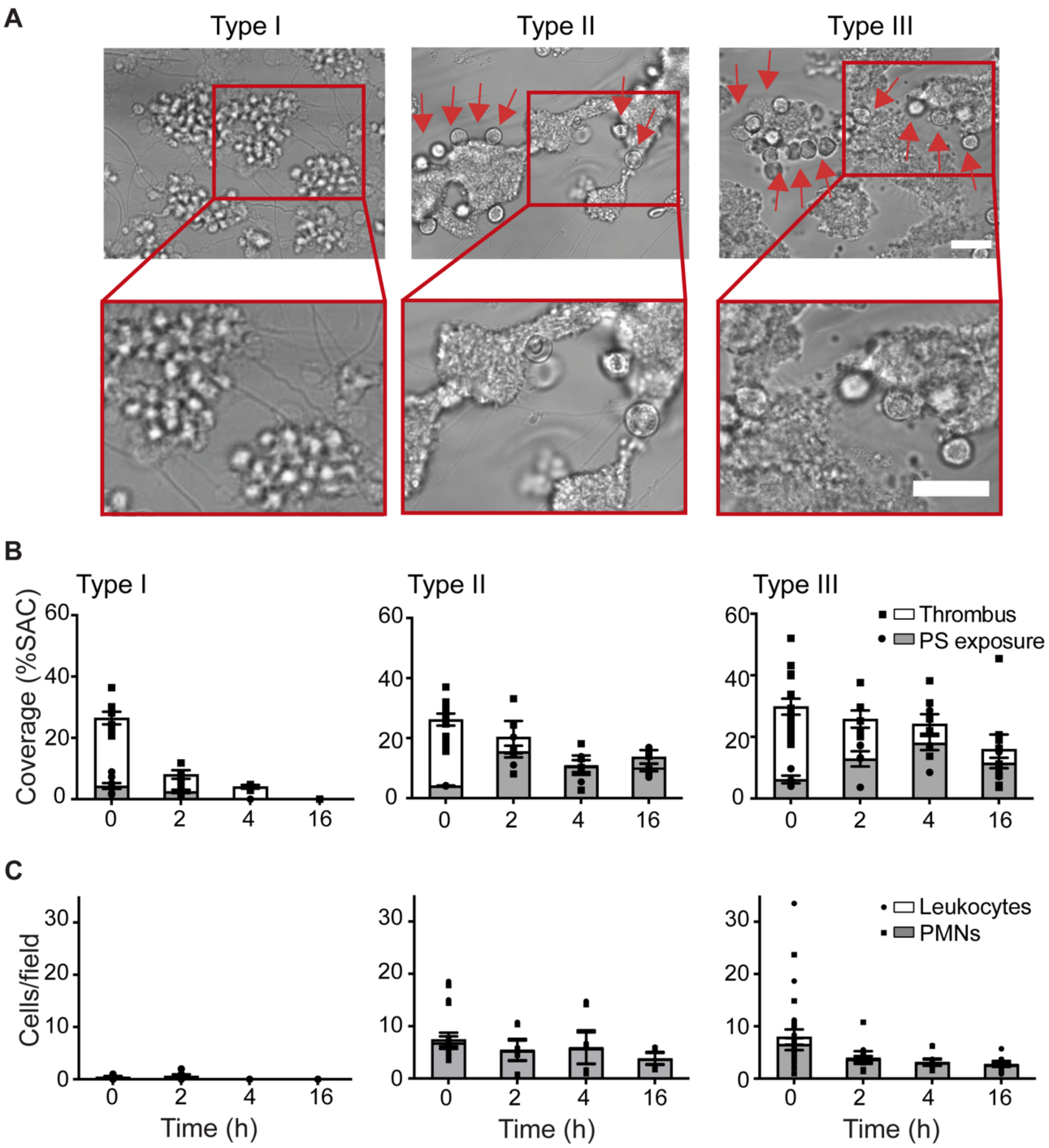

Figure S3. Long-term leukocyte adhesion at thrombi with highly activated platelets

Whole blood was flowed over collagen in a microfluidic chamber at 1000 s^{-1} for 4 minutes to form thrombi of types I-III. *Type I*: perfusion of PPACK-anticoagulated blood; *type II*: co-perfusion of PPACK-anticoagulated blood in the presence of Me-SADP (1 μmol/L, f.c.); *type III*: co-perfusion of citrated blood with coagulation medium containing tissue factor and $CaCl_2/MgCl_2$. Leukocyte adhesion was induced by 10 seconds of stasis and 2 minutes of additional flow. Chambers were observed by brightfield and fluorescence microscopy after 0, 2 or 16 hours at 37°C, after which 5 random multicolor images were taken. **A**, Representative images of type I-III thrombi, as well as enlargements. Arrows indicate adhered leukocytes (bar, 25 μm). **B**, Surface area coverage (%SAC) of thrombi and activated, phosphatidylserine-exposing platelets (AF647 annexin A5) at collagen surface. **C**, Counts per microscopic field 1.49 mm^2 (141×106 μm) of total adhered leukocytes (brightfield) and identified polymorphonuclear cells (PMNs) (AF647 anti-CD15 mAb). Mean±SE (n=4-8)

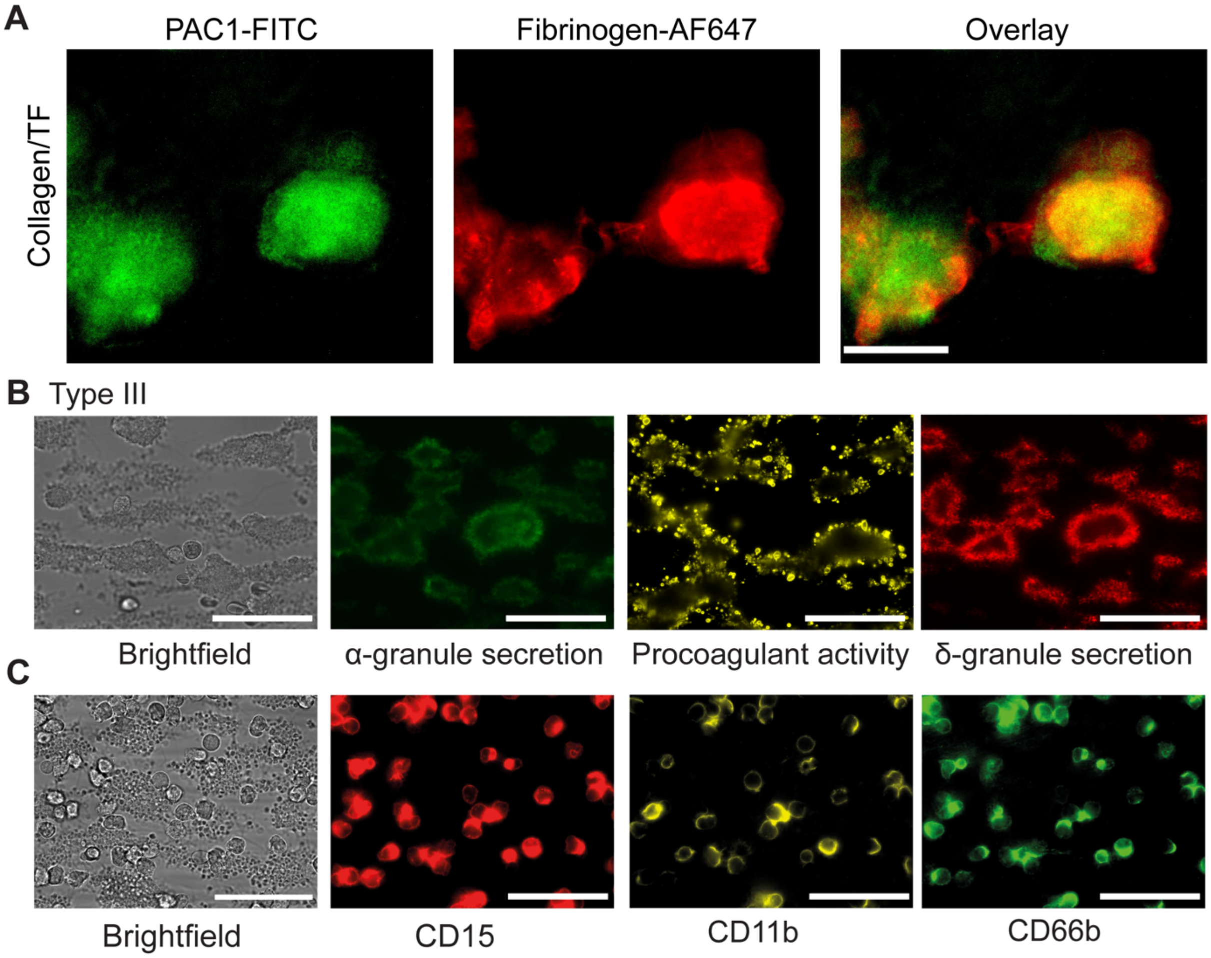

Figure S4. Platelet activation and leukocyte characterization of type III thrombi

Whole blood was flowed over collagen under coagulant conditions at 1000 s^{-1} for 4 minutes to form type III thrombi with leukocytes (see Figure S3). **A**, Overlay of thrombi stained with FITC PAC1 mAb and AF647 fibrinogen, showing activated αIIbβ3 integrin as fibrinogen-binding receptors. **B**, Representative brightfield and multicolor fluorescence images after staining for secretion of α-granules (FITC anti-CD62P mAb) and δ-granules (AF647 anti-CD63 mAb), and for phosphatidylserine exposure (AF568-annexin A5). Brightfield and tricolored fluorescence image are from the same field of view, but not overlapping due to different optics. Note secretory platelets in thrombi and distinct, single phosphatidylserine-exposing platelets edging around the thrombi. **C**, Post-staining of thrombi for neutrophils ($CD15^+$, AF647 anti-CD15 mAb) as well as activation markers using PE anti-CD11b mAb and FITC anti-CD66b mAb (all 2 µg/mL). Shown are representative overlay images (n=3); bar, 25 µm.

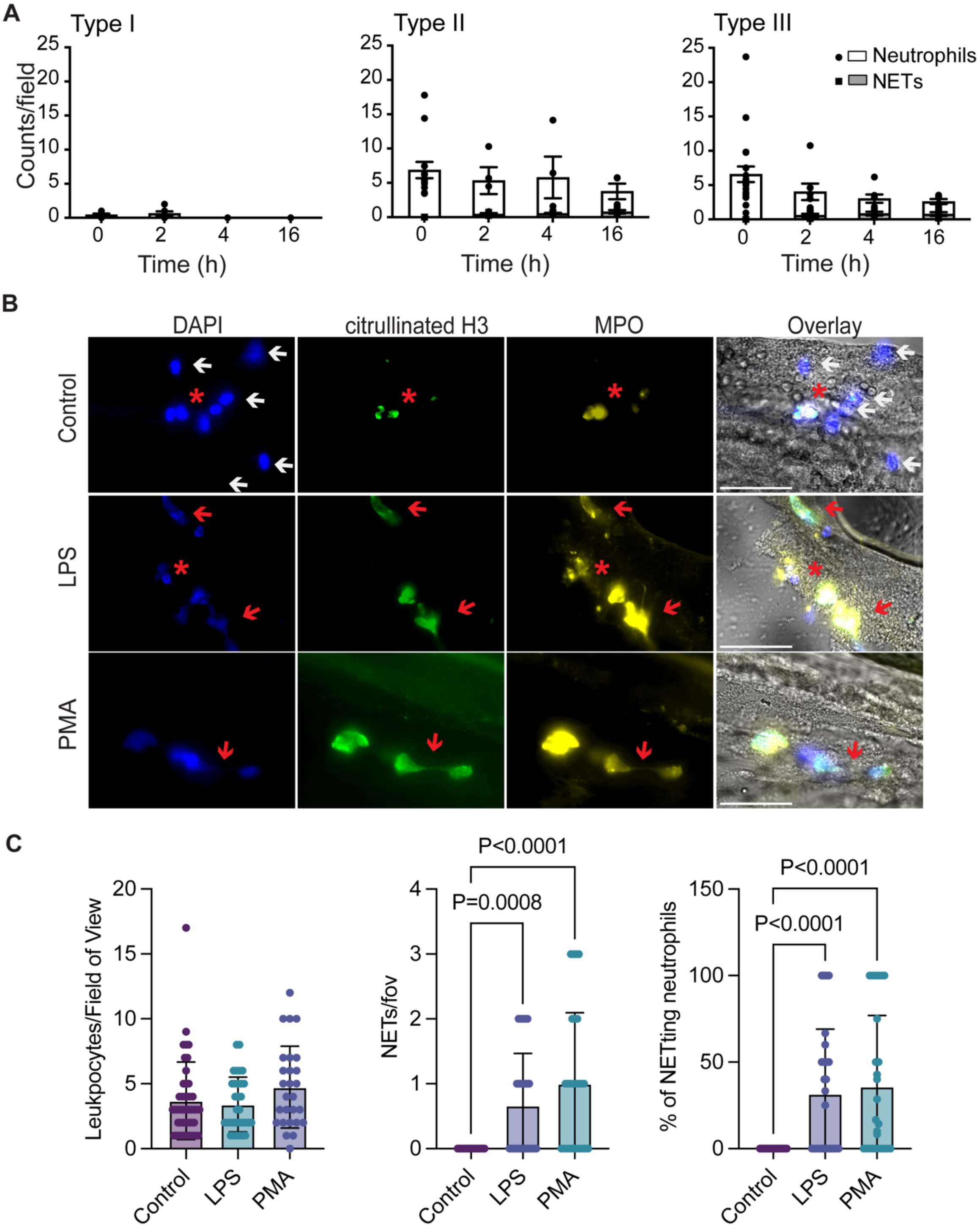

Figure S5. Activation and NET formation of neutrophils adhered to thrombi

Thrombi of type I-III with adhered leukocytes were formed in microfluidic chambers and incubated for 0-16 h at 37°C in incubation medium containing antibiotics, as for Figure S3. **A**, Counts of leukocytes and NETs per microscopic field. Mean±SE (n=3-8). Presence of NETs was concluded from cells showing a distorted nucleus (DAPI staining). **B**, Stimulation of neutrophil NET formation on thrombi by incubation with 50 nmol/L PMA or 5 μg/mL LPS for 4 hours. **B,** Representative microscopic images, bar = 50 μm,**C,** Quantification of NETs after 4 hours of incubation (n=28 cells, from 3 independent experiments, Kruskal-Wallis test with Dunn's multiple comparison), as observed from co-staining of DAPI and citrullinated histone.

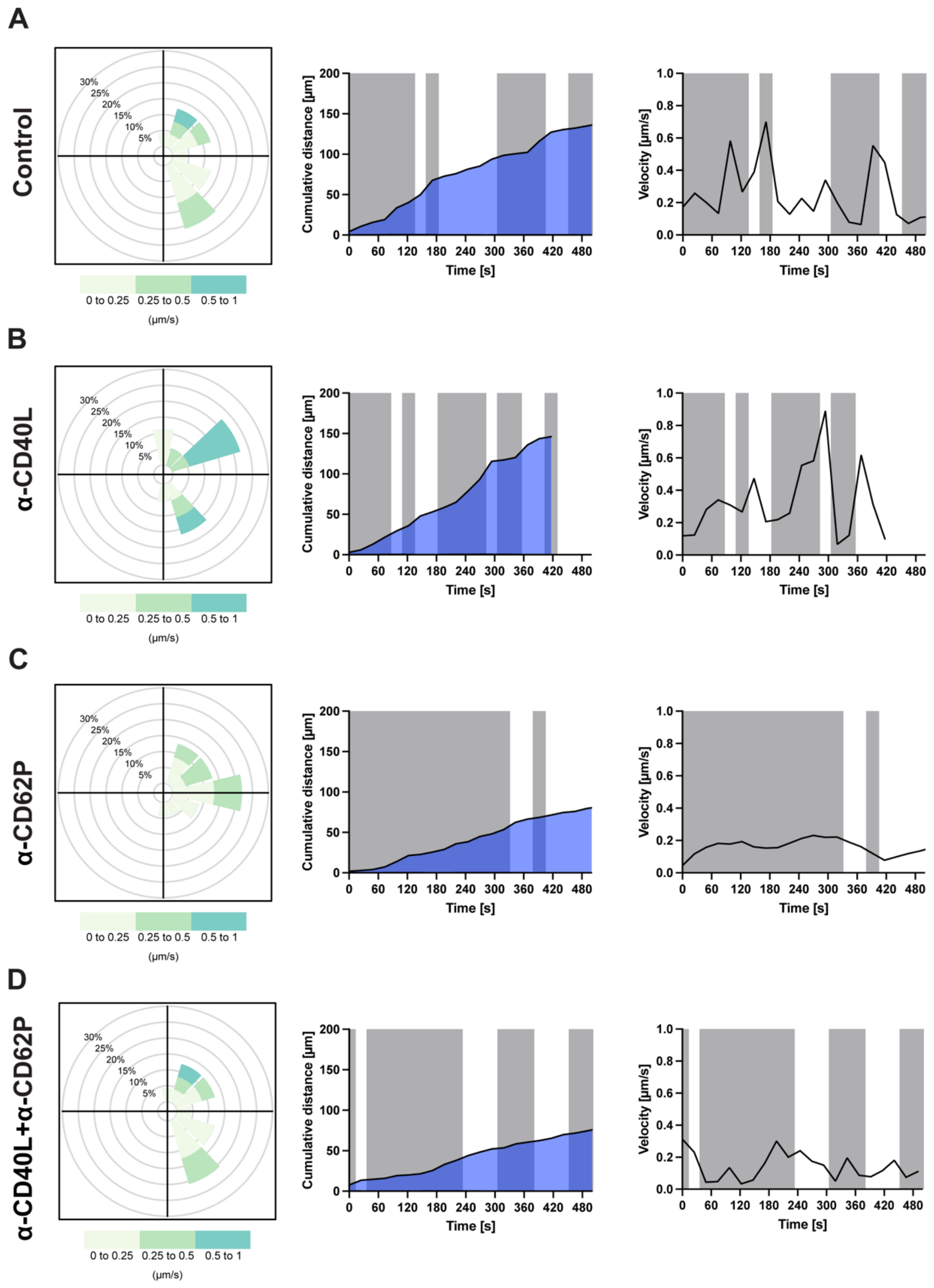

Figure S6. Inhibition of CD40L increases leukocyte movement at and around thrombi

A, Rose plot showing analysis of leukocyte movement at and around the thrombi, as well as cumulative distance and velocity under control condition. **B**, Idem in the presence of blocking anti-CD40L mAb; **C,** in the presence of blocking anti-CD62P mAb; and **D,** with both antibodies present. Note that the wind Rose plots show movement of one representative cell per condition. For the same cells, the cumulative distance over time is shown (middle graph), and the velocity of movement (i.e. first derivative).

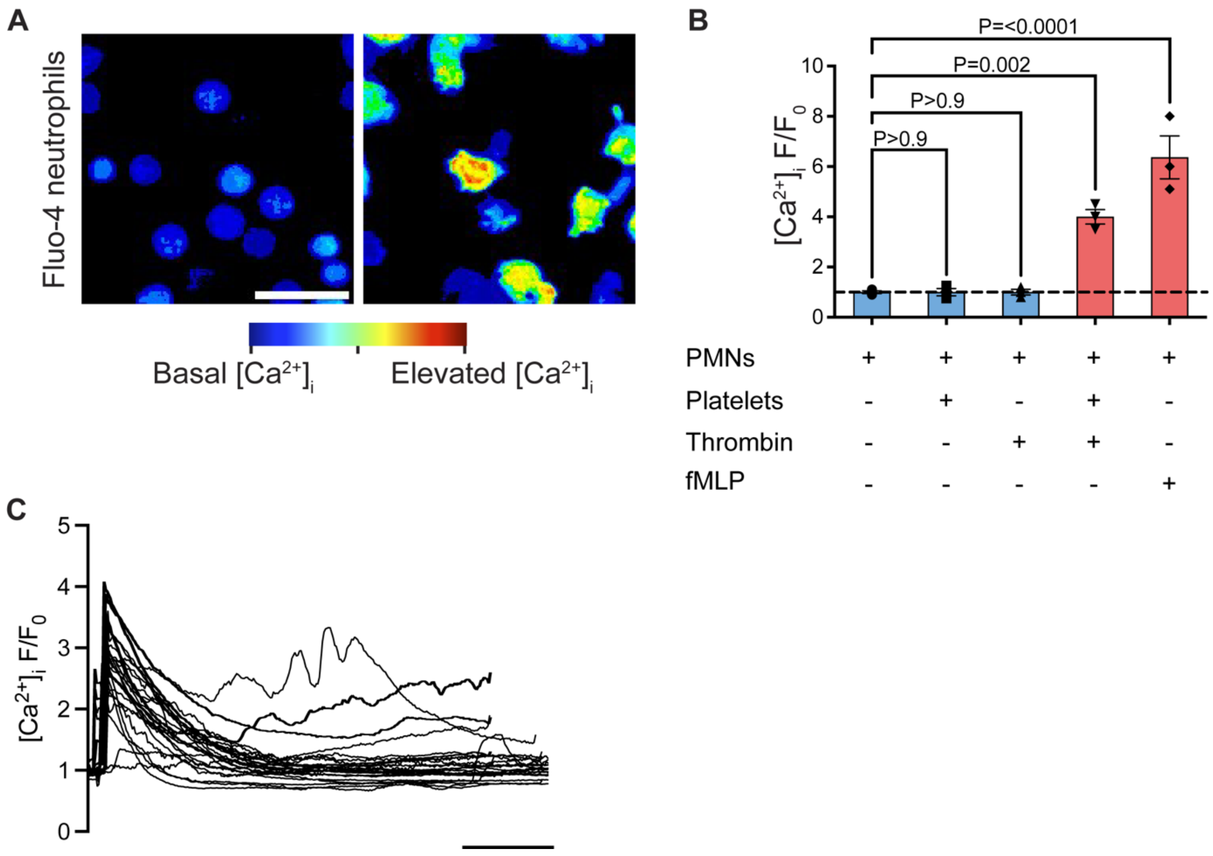

Figure S7. Activation of platelets inducing leukocyte Ca^{2+} rises

A-C, Fluo-4-loaded granulocytes ($1×10^6$/mL) in wells were allowed to adhere under stasis, and rises in $[Ca^{2+}]_i$ (*F/F₀*) were measured by microscopic imaging for 10 minutes. Added were platelets ($10×10^7$/mL), iloprost (10 nmol/L), thrombin (4 nmol/L) and/or fMLP (1 µmol/L), as indicated. **A**, Representative Fluo-4 images before (left) and after (right) fMLP addition. The fMLP calcium responses indicative for presence of neutrophils. Scale bar 25 µm **B**, Quantification of maximal rises in $[Ca^{2+}]_i$ (>10 cells) per condition. Mean±SE (n=3), 1-way ANOVA. **C**, Single $[Ca^{2+}]_i$ traces of cells adhered to a coverslip induced by fMLP(1 µmol/L), summed from three independent experiments. Bar = 1 min.

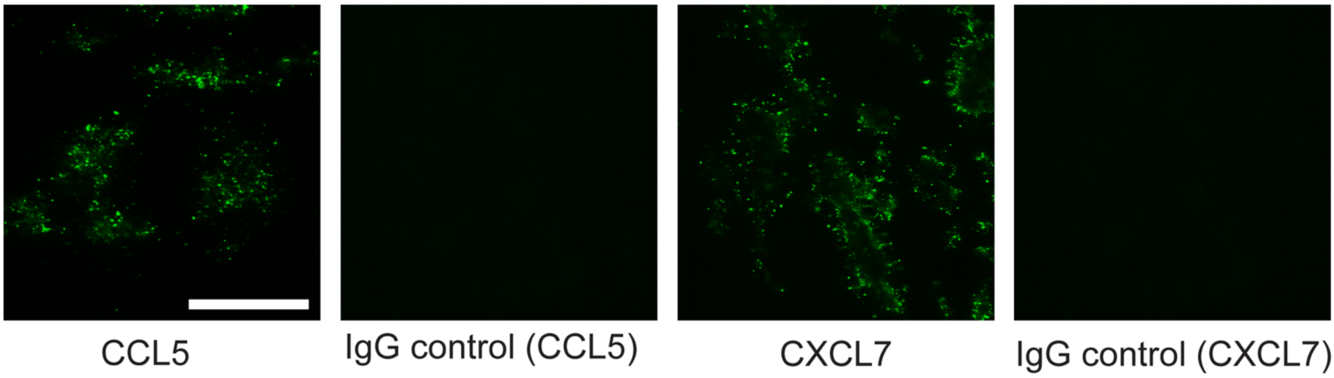

Figure S8. Cytokine expression on thrombi of activated platelets

Whole blood was flowed over collagen under coagulant conditions at 1000 s^{-1} for 4 minutes to form type III thrombi. Released CCL5 was detected with a rabbit polyclonal antibody (Abcam, ab9679), released CXCL7 with rabbit anti-NAP2 polyclonal antibody (Proteintech, 13313). Visualization was with FITC donkey anti-rabbit IgG at 5 μg/mL. Staining with IgG controls is shown as well. Representative confocal fluorescence images (n=3); bar, 25 μm.

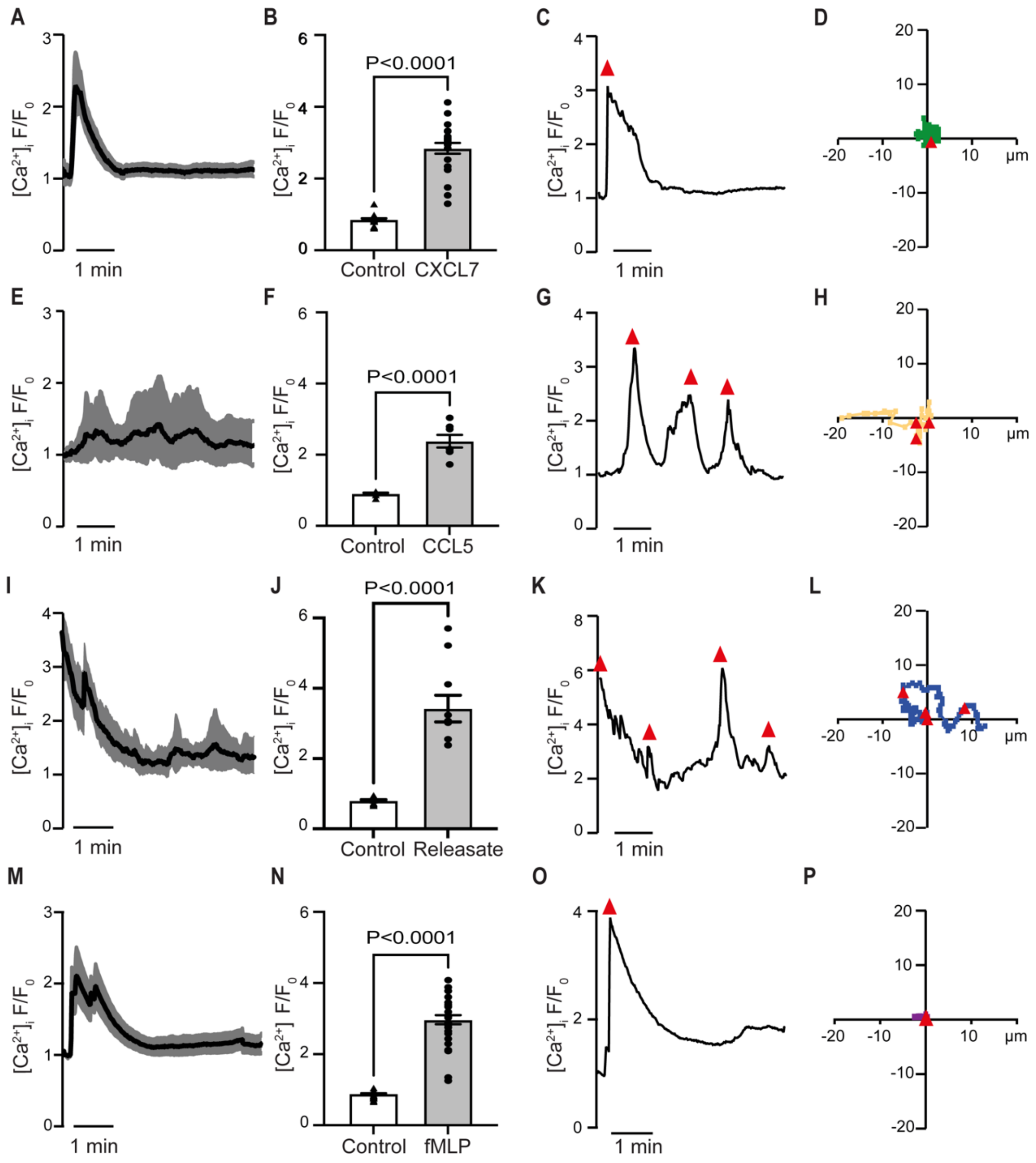

Figure S9. Distinct chemokine- and releasate-induced Ca^{2+} responses in isolated leukocytes

Fluo-4-loaded granulocytes (1×10^6/mL) in wells were allowed to adhere to coverslips under stasis, and rises in $[Ca^{2+}]_i$ (F/F_o) were measured by microscopic imaging for 5 minutes. Added to the wells were Hepes buffer (control), CXCL7 (100 ng/mL), CCL5 (100 ng/mL), or fMLP (4 μmol/L). Alternatively, the centrifuged releasate from pre-activated washed platelets was added. Transient rises in $[Ca^{2+}]_i$ and chemotactic movement were evaluated for 5 minutes by brightfield and fluorescence video-imaging. Shown (from left to right) are chemokine-induced integrated Ca^{2+} responses, quantified maximal $[Ca^{2+}]_i$ rises; and from a representative neutrophil both the $[Ca^{2+}]_i$ transient and the movement pattern during 5 minutes. Red arrow heads indicate times of Ca^{2+} peaks. **A-D**, Effect of CXCL7 addition. **E-H**, Effect of CCL5 addition. **I-L**, Effect of platelet releasate. **M-P**, Effect of fMLP, indicative of neutrophil responsiveness. Mean±SE (n=7-33 cells from 3 independent experiments) unpaired t-test.

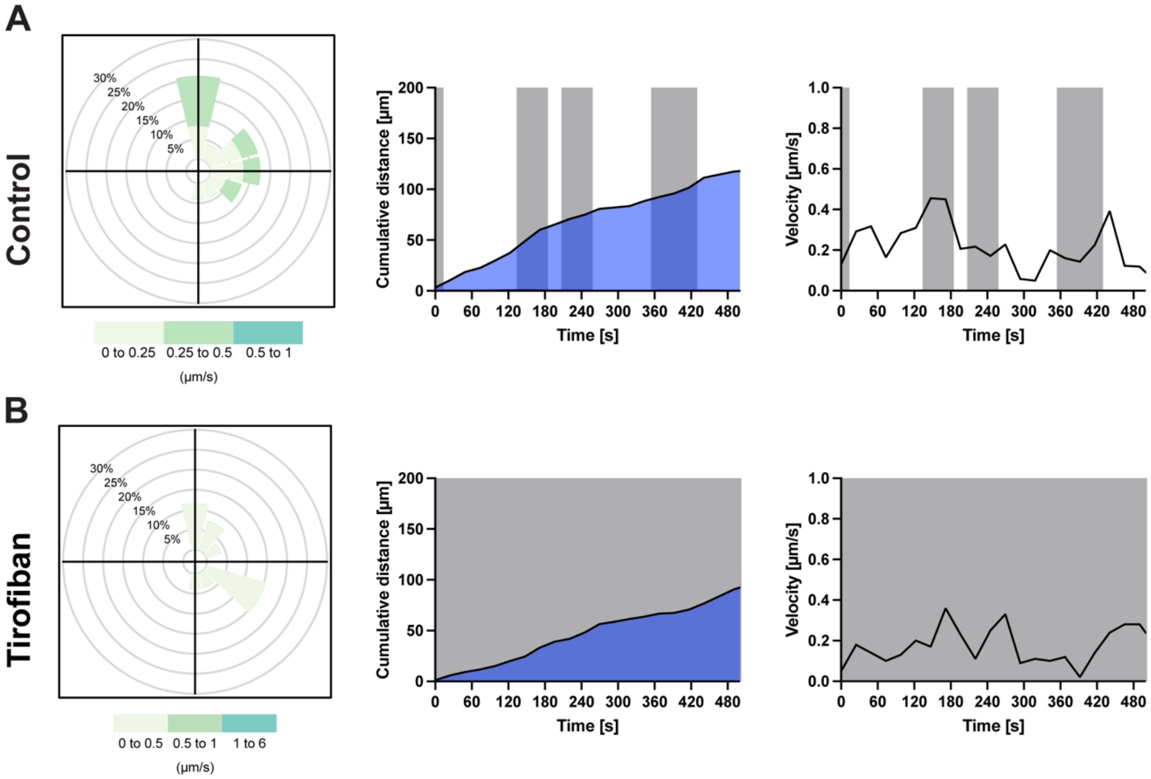

Figure S10. Blocking of integrin αIIbβ3 reduces leukocyte movement on thrombi

Movement analysis of representative neutrophils on thrombi in **(A)** control conditions and **(B)** with inhibition platelets integrin αIIbβ3 (1 µM tirofiban). Shown are rose plots, the cumulative distance over time and the neutrophil velocity over time. Representative graphs.

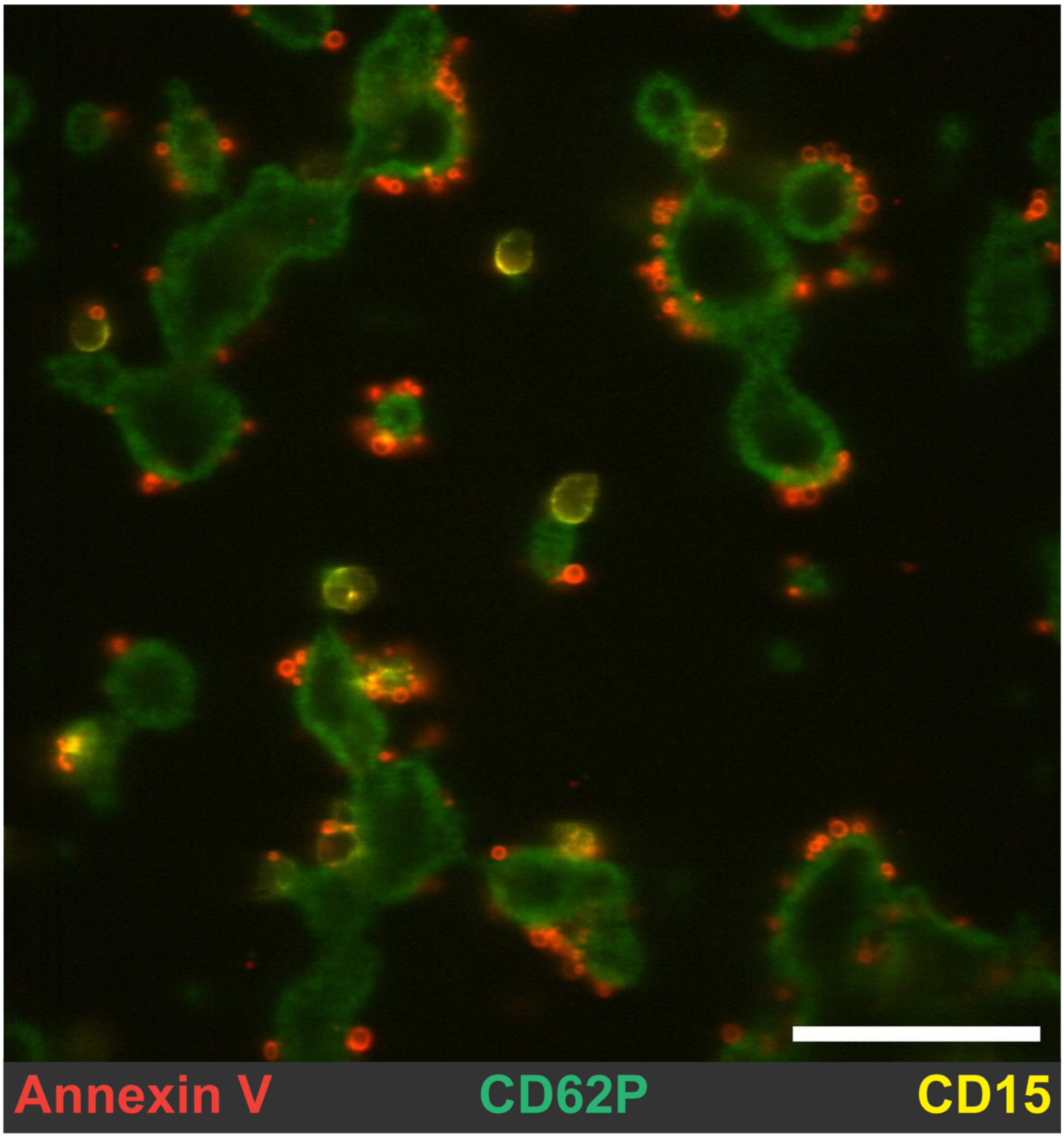

Figure S11. Leukocytes on platelets expressing CD62P and phosphatidylserine

Whole blood was flowed over collagen under coagulant conditions at 1000 s^{-1} for 4 minutes to form type III thrombi. The thrombi with leukocytes (in majority neutrophils) were post-perfused with labels AF647 annexin A5, FITC anti-CD62P mAb and AF568 anti-CD15 mAb. Shown is the still of a representative tricolored fluorescence movie, n=3 (see Online Video 3). Note the partial overlay of red and yellow colors. Scale bar 50 µm.

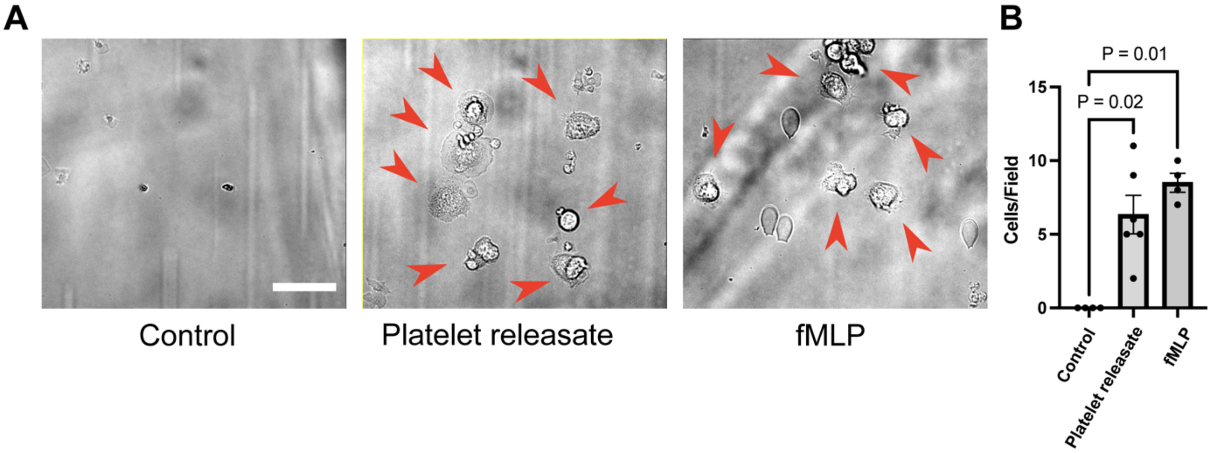

Figure S12. Platelet releasate and fMLP inducing leukocyte adhesion to fibrinogen

A, B, Isolated granulocytes (fixed count, in majority neutrophils) were flowed over a fibrinogen surface at low shear rate of 200 s^{-1} for 10 minutes, and numbers of attached cells per microscopic field were counted. Added then was releasate from CRP-XL- plus TRAP6-activated platelets (10 vol%) or fMLP (1 μmol/L). **A**, Representative brightfield microscopic images. Bar, 25 μm. **B**, Cell counts per field. Note the similarity of platelet releasate and fMLP to cause cell (neutrophil) adhesion Mean±SE (n=4-6), 2-way-ANOVA.

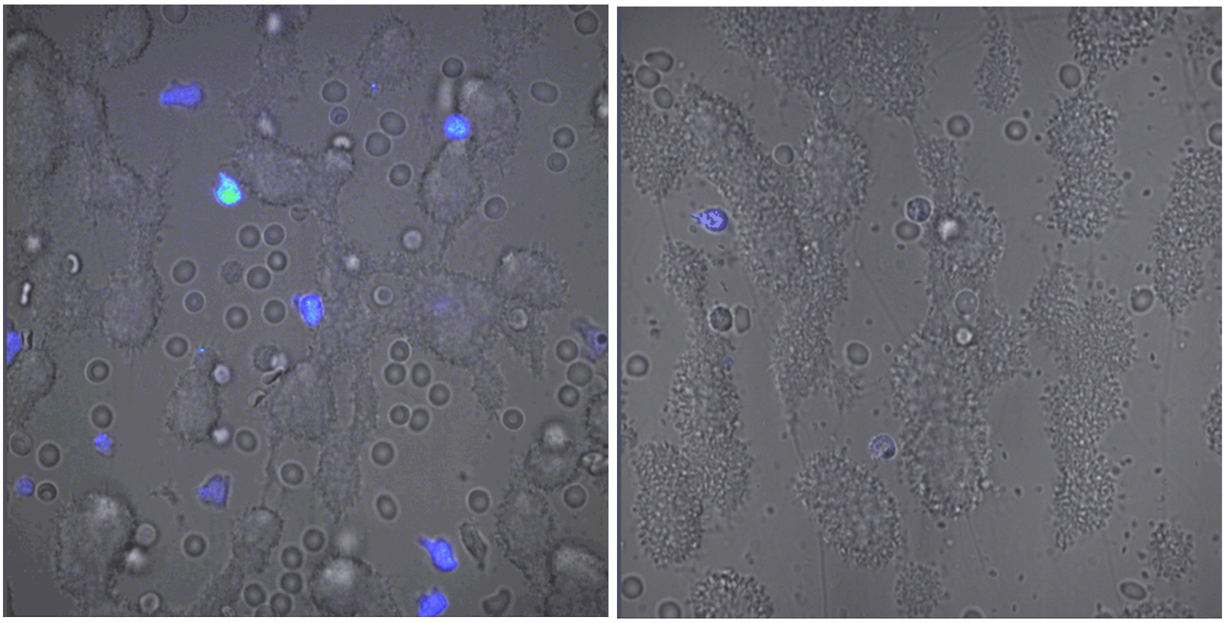

Online videos S1 and S2. Type III thrombi containing leukocytes were left untreated (control, left) or post-perfused with iloprost (10 nM, right video) for 10 min. Changes in $[Ca^{2+}]_i$ in Fluo-4-loaded cells adhered to thrombi were measured. Recorded were 10-min fluorescence and brightfield microscopic movies. Image sizes 213 x 213 µm.

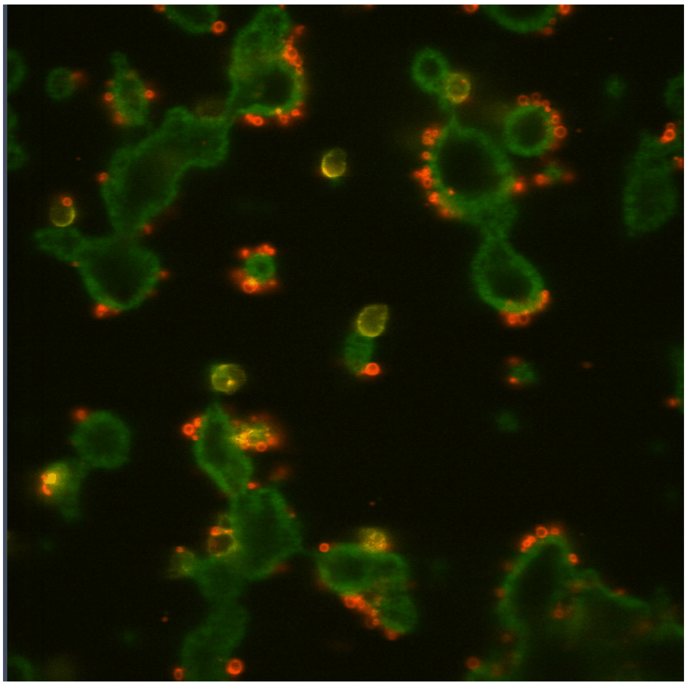

Online video S3. Whole blood was flowed over collagen under coagulant conditions at 1000 s^{-1} for 4 min to form type III thrombi (see Figure S3). Thrombi were then post-perfused with AF647-labelled annexin A5, FITC-anti-CD62P mAb and AF568-anti-CD15, and imaged for 10 minutes. Image size 213 x 213 µm.

Data table S1

Parameter	Healthy controls	GT patients
WBC (10^9/L)	4.83± 0.21	6.77±1.85
RBC (10^{12}/L)	4.36±0.27	4.31±0.49
HGB (g/L)	129.33±10.21	126.00±15.72
HCT (%)	38.03±3.78	38.97±5.86
PLT (10^9/L)	236.67±46.07	174.33±42.25

Table S1. Haematologic parameters are similar between Glanzmann thrombasthenia patients and healthy controls. WBC = white blood cells, RBC = red blood cells, HGB = haemoglobin, HCT = haematocrit, PLT = platelets. Data previously published by Van Geffen et al. (Haematologica. 2019; 104: 1256-126).

Major Resources Table

Animals (in vivo studies)

Species	Vendor or Source	Background Strain	Sex	Persistent ID / URL
N/A	N/A	N/A	N/A	N/A

Genetically Modified Animals

	Species	Vendor or Source	Background Strain	Other Information	Persistent ID / URL
**Parent - Male**	N/A	N/A	N/A	N/A	N/A
**Parent - Female**	N/A	N/A	N/A	N/A	N/A

Antibodies

Target antigen	Vendor or Source	Catalog #	Working concentration	Lot # (preferred but not required)	Persistent ID / URL
AF647 anti-human CD62P mAb	BioLegend (San Diego, CA, USA)	304918	0.5 μg/mL	n.d.	https://www.biolegend.com/en-us/products/alexa-fluor-647-anti-human-cd62p-p-selectin-antibody-3348
APC anti-CD63 mAb	eBioscience (San Diego, CA, USA)	17-0631-82	1.5 μg/mL	n.d.	https://www.thermofisher.com/ antibody/ product/CD63-antibody-clone-NVG-2-monoclonal/17-0631-82
Donkey anti-rabbit IgG	Invitrogen (Carlsbad, CA, USA)	A166025	5 μg/mL	n.d.	https://www.thermofisher.com/ antibody/ product/Donkey-anti-Rabbit-IgG-H-L-Secondary-Antibody-Polyclonal/A16025
Rabbit anti-CXCL7 (NAP2) pAb	Sanbio (Uden, NL)	A304-579A-T	1 μg/mL	n.d.	https://www.sanbio.nl/a304-579a-t
Rabbit anti-CCL5 pAb	Abcam (Cambridge, UK)	ab9679	1 μg/mL	n.d.	https://www.abcam.com/rantes-antibody-ab9679
Rabbit anti-CXCL4 mAb	Abcam (Cambridge, UK)	ab129183	1 μg/mL	n.d.	https://www.abcam.com/pf4-antibody-epr7763-ab129183
ChIP grade rabbit anti-human H3 citrulline histone pAb	Abcam (Cambridge, UK)	ab5103	1 μg/mL	n.d.	https://www.abcam.com/histone-h3-citrulline -r2--r8--r17-antibody-ab5103
FITC anti-CD66b mAb	BioLegend (San Diego, CA, USA)	305104	1-5 μg/mL	n.d.	https://www.biolegend.com/en-us/products/fitc-anti-human-cd66b-antibody-666
AF647 anti-CD15 mAb	BioLegend (San Diego, CA, USA)	125608	2 μg/mL	n.d.	https://www.biolegend.com/en-us/products/alexa-fluor-647-anti-mouse-human-cd15-ssea-1-antibody-4819
Anti-LFA1 mAb	BioLegend (San Diego, CA, USA)	363412	5 μg/mL	n.d.	https://www.biolegend.com/en-us/products/alexa-fluor-647-anti-human-cd11acd18-lfa-1-antibody-14972

PE anti-CD11b mAb	BioLegend (San Diego, CA, USA)	301306	2 μg/mL	B279251	https://www.biolegend.com/en-us/products/pe-anti-human-cd11b-antibody-768
Anti-CD162 mAb	BioLegend (San Diego, CA, USA	328806	2 μg/mL	B270016	https://www.biolegend.com/en-us/products/pe-anti-human-cd162-antibody-4510
FITC anti-CD62P mAb	Beckman Coulter (Brea, CA, USA)	07790	2 μg/mL	20051	https://www.beckman.de/reagents/coulter-coulter-flow-cytometry/antibodies-and-kits/single-color-antibodies/cd62p/a07790
AF647 anti-CD63 mAb	R&D Systems (Minneapolis, MN, USA)	IC5048R	2 μg/mL	n.d.	https://www.rndsystems.com/products/human-cd63-alexa-fluor-647-conjugated-antibody-460305_ic5048r
PE anti-MPO mAb	eBioscience (San Diego, CA, USA)	11-1299-41	3 μg/mL	n.d.	https://www.thermofisher.com/antibody/product/myeloperoxidase-MPO-antibody-clone-MPO455-8E6-monoclonal/11-1299-41
PerCP anti-CD42a mAb	BD Biosciences (Haryana, India)	340537	2 μg/mL	n.d.	https://www.bdbiosciences.com/en-ca/products/reagents/flow-cytometry-reagents cd42a.340537
PE anti-CD41a mAb	ThermoFisher (Eindhoven, NL)	12-0419-42	1 μg/mL	n.d.	https://www.thermofisher.com/antibody/ product/CD41a-Antibody-clone-HIP8-monoclonal/12-0419-42
FITC PAC1 mAb	ThermoFisher (Eindhoven, NL)	MA5-28564	2 μg/mL	n.d.	https://www.thermofisher.com/antibody/ product/PAC-1-antibody-clone-PAC-1-monoclonal/MA5-28564
AF647 anti-CD15 mAb	BioLegend (San Diego, CA, USA)	125608	5 μg/mL	n.d.	https://www.biolegend.com/en-us/products/alexa-fluor-647-anti-mouse-human-cd15-ssea-1-antibody-4819

DNA/cDNA Clones

Clone Name	Sequence	Source / Repository	Persistent ID / URL
N/A	N/A	N/A	N/A

Cultured Cells

Name	Vendor or Source	Sex (F, M, or unknown)	Persistent ID / URL
N/A	N/A	N/A	N/A

Data & Code Availability

Description	Source / Repository	Persistent ID / URL
N/A	N/A	N/A

Other

Description	Source / Repository	Persistent ID / URL

DiOC6	ThermoFisher (Eindhoven, NL)	https://www.thermofisher.com/order/catalog/product/DiOC6
Fluo-4 acetoxy methyl ester	ThermoFisher (Eindhoven, NL)	https://www.thermofisher.com/order/catalog/product/F14201
Pluronic	Invitrogen (Carlsbad CA, USA)	https://www.thermofisher.com/order/catalog/product/P3000MP
D-Phe-Pro-Arg chloromethyl ketone (PPACK)	Santa Cruz Biotechnology (Santa Cruz, CA, USA)	https://www.scbt.com/p/ppack-dihydrochloride
Human fibrinogen	Enzyme Research Laboratories (Swansea, UK)	https://www.enzymeresearch.co.uk/product/human-fibrinogen--von-willebrand-factor-and-plasminogen-depleted
Human alpha-thrombin	Enzyme Research Laboratories (South Bend, IN, USA)	https://enzymeresearch.com/product/human-alpha-thrombin
Collagen-I Horm	Takeda (Chou-ku, Osaka, Japan	https://www.takeda.com/de-at/hcps/diagnostika Kollagen reagens Horm
Human collagen-III	Southern Biotechnology (Birmingham, AL, USA)	https://www.southernbiotech.com/human-type-iii-collagen
Tissue factor (Innovin)	ThermoFisher (Eindhoven, NL)	https://www.fishersci.com/shop/products/dade-innovin-pt-reagent-us-3/p-7235905
4',6-Diamidino-2-phenylindole (DAPI)	Life Technologies (Carlsbad, CA, USA)	https://www.thermofisher.com/order/catalog/product/D1306
Alexa Fluor (AF) 488 (568) annexin A5	ThermoFisher (Eindhoven, NL)	https://www.thermofisher.com/order/catalog/product/A23204
Recombinant human CCL5	R&D Systems (Minneapolis MN, USA)	https://www.rndsystems.com/products/recombinant-human-ccl5-rantes-protein_278-rn
Penicillin/Streptomycin	Thermo-Fisher Scientific (Eindhoven, NL)	https://www.fishersci.de/shop/products/gibco-penicillin-streptomycin-10-000-u-ml -3/11548876
Phorbol-12-myristate-13-acetate (PMA)	Sigma-Aldrich (St. Louis, MO, USA)	https://www.fishersci.de/shop/products/gibco-penicillin-streptomycin-10-000-u-ml-3/11548876
Histopaque-1077 / 1119	Sigma-Aldrich (St. Louis, MO, USA)	https://www.sigmaaldrich.com/NL/en/product/sigma/10771 https://www.sigmaaldrich.com/NL/en/product/sigma/11191
Iloprost	Bayer Schering Pharma (Berlin, Germany)	https://pharma.bayer.nl/ iloprost

2-Methylthio (Me-S)-ADP	Santa Cruz Biotechnology (Santa Cruz, CA, USA)	https://www.scbt.com/p/methyl-2-methylthio-butyrate-51534-66-8
2',7'-Dichlorodihydro fluorescein diacetate (H2DCFDA)	Thermo-Fisher Scientific (Eindhoven, NL)	https://www.thermofisher.com/order/catalog/product/D399
Sytox Green	Thermo-Fisher Scientific (Eindhoven, NL)	https://www.thermofisher.com/order/catalog/product/R37168
CRP-XL	Cambcol Laboratories	https://www.cambcollabs.com/ #CRP-XL
CXCL7	Peprotech (London UK)	https://www.peprotech.com/en/recombinant-human-nap-2-cxcl7
fMLP	Merck (Darmstadt, Germany)	https://www.merckmillipore.com/DE/de/product/Formyl-Met-Leu-Phe-OH-CAS-59880-97-6-Calbiochem
Cell-ROX Deep Red	Thermo-Fisher Scientific (Eindhoven, NL)	https://www.thermofisher.com/order/catalog/product/C10422
Gly-Pro-Arg-Pro peptide (GPRP)	Thermo-Fisher Scientific (Eindhoven, NL)	https://www.fishersci.com/shop/products/h-gly-pro-arg-pro-nh-5-mg/50382505
Thrombin receptor-activating peptide 6 (TRAP6)	Bachem (Bubendorf, Switzerland)	https://shop.bachem.com/product/4017752
Trisodium citrate	Sigma-Aldrich (St. Louis, MO, USA)	https://www.sigmaaldrich.com/NL/en/product/aldrich/w302600
AF647 fibrinogen	Thermo-Fisher Scientific (Eindhoven, NL)	https://www.thermofisher.com/order/catalog/product/F35200?SID=srch-srp-F35200
